# Marine Bromophenols from *Laminaria hyperborea*’s Epiphytic Biomass: Chemical Profiling, Cytotoxicity, and Antioxidant Activity

**DOI:** 10.3390/md24010052

**Published:** 2026-01-21

**Authors:** Angeliki Barouti, Vinh Le Ba, Lars Herfindal, Monica Jordheim

**Affiliations:** 1 Department of Chemistry, Faculty of Natural Sciences and Technology, University of Bergen, 5007 Bergen, Norway; angeliki.barouti@uib.no (A.B.); vinh.ba@uib.no (V.L.B.); 2 Centre for Pharmacy, Department of Clinical Science, Faculty of Medicine, University of Bergen, 5009 Bergen, Norway; lars.herfindal@uib.no

**Keywords:** epiphytes, red algae, *Laminaria hyperborea*, bromophenols, cytotoxicity, leukemia, zebrafish model, antioxidant activity, marine biorefinery, sustainability

## Abstract

The epiphytic community of *Laminaria hyperborea*, dominated by red algae, is typically discarded during industrial processing despite its potential as a source of high-value natural products. This study aims to valorize this underutilized biomass by characterizing its secondary metabolites and evaluating the biological activities of its major bromophenols. A combined chromatographic workflow enabled the isolation and structural elucidation of five bromophenols (**1**–**5**), including one previously undescribed compound (**5**). Among these, compound **4** exhibited the strongest cytotoxicity against the acute myeloid leukemia (AML) cell line MOLM-13 (EC_50_ = 6.23 μM) and induced pronounced apoptotic features. When tested on two normal cell lines (NRK and H9c2) and in zebrafish larvae, it showed moderate toxicity at higher concentrations, indicating a reasonable selectivity window. In contrast, compound **5** was more toxic to normal cells than to MOLM-13 in vitro, while showing no acute toxicity in zebrafish; however, interpretations are preliminary due to compound purity. Bromophenols **1**–**4** were also tested for antioxidant activity, with **4** being the most potent (ABTS EC_50_ = 22.1 μM), although this did not translate into protection against doxorubicin-induced cardiotoxicity. Additionally, non-targeted UHPLC-QTOF MS/MS analysis tentatively identified nine additional bromophenols and provided an estimation of their origin species within the epiphytic assemblage.

## 1. Introduction

Marine ecosystems represent one of the most chemically diverse yet underexplored sources of natural products. Marine algae are recognized for producing a wide array of structurally unique metabolites with significant biological potential. Among these, halogenated compounds, especially brominated derivatives, stand out, since bromination occurs almost exclusively in marine environments due to the high bromide concentration in seawater. Brominated compounds were first reported in algae in the early 1900s, and since then, numerous structures have been isolated and investigated for their biological properties [1].

Within this group, bromophenols (BPs) have attracted considerable attention. These compounds typically consist of one or more benzene rings with varying numbers of hydroxyl, bromine, and/or other substituents [2]. Bromophenols are most abundant in red algae but are also found in green and brown algae, and occasionally in marine animals and fungi [3].

Over the years, several studies have demonstrated that marine bromophenols exhibit various biological activities in vitro and in vivo, including anticancer, antioxidant, anti-inflammatory, antidiabetic, and antimicrobial effects [2,4]. Consequently, they have emerged as promising candidates for applications in pharmaceuticals, nutraceuticals, and functional foods, with possible roles in managing cancer, cardiovascular diseases, neurodegeneration, inflammation, diabetes, and microbial infections [2,4].

Despite this promise, research on bromophenols faces several challenges. Their natural abundance is generally low, limiting the quantities available for structural characterization and biological evaluation. For example, in the case of *Rhodomela confervoides*, more than 15 kg of dried material is required to obtain sufficient amounts of bromophenols [5,6,7]. As a result, traditional bioprospecting, harvesting marine organisms, raises ethical, ecological, and sustainability concerns [8]. Furthermore, the chemical profile of algae can vary substantially with location and season [9], introducing additional uncertainty on the yield and composition of bioactive metabolites. Collectively, these factors hinder the practical exploitation of marine bromophenols.

These limitations underscore the need for sustainable strategies, and mariculture now plays an increasingly important role in addressing this demand. Norway, which harbors one of the largest standing kelp biomasses globally [10], has a long tradition of seaweed harvesting and utilization. Each year, more than 150,000 wet tons of *Laminaria hyperborea* are collected from regulated harvesting areas, ensuring both a reliable biomass supply and ecological balance [10,11]. The kelp stipe hosts a rich epiphytic community dominated by red algae, along with small invertebrates that use its surface as a substrate. Our previous work has shown that the dominant epiphytic species include *Palmaria palmata*, *Rhodomela* sp., *Ptilota gunneri*, and *Membranoptera alata*, along with smaller amounts of other red algae [8]. Many of these species are known or expected to produce diverse metabolites with possible bioactivities. Despite this potential, during industrial processing, the epiphytic biomass—around 7500 tons annually—is removed and treated as waste [8].

The novelty and aim of this study are to valorize this underutilized epiphytic biomass as a source of high-value compounds, particularly bromophenols. Five bromophenols (**1**–**5**) were isolated from the ethyl acetate fraction using various chromatographic techniques, and their structures were elucidated by mass spectrometry and NMR spectroscopy. Notably, compound **5** was identified as a previously undescribed bromophenol, adding to the chemical diversity of brominated metabolites. The compounds were evaluated for cytotoxicity against leukemia cells, and two normal cell lines in vitro, as well as zebrafish larvae in vivo. Antioxidant assays were performed on the major bromophenols **1**–**4** to examine the relationship between radical-scavenging capacity and cytotoxic behavior. Given the pronounced antioxidant activity of **4**, its protective effect against doxorubicin-induced cardiotoxicity was further investigated. Finally, HRMS-QTOF analysis was employed to characterize the broader bromophenolic profile of the epiphytes, tentatively identify an additional nine candidate metabolites, and trace their origin to the individual algal species, including *Rhodomela lycopodioides*, whose chemistry has not previously been described.

## 2. Results

### 2.1. Isolation and Characterization

**Figure 1:**
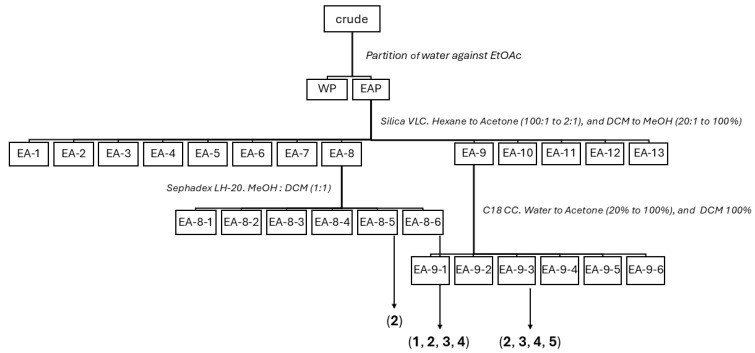
Workflow illustrating the extraction and isolation process for bromophenols **1**–**5**.

To efficiently identify the bromophenols present in the epiphytic biomass of *L. hyperborea* and evaluate their biological potential, a stepwise workflow was applied, combining solvent partitioning, chromatographic separation, ^1^H NMR/MS-guided detection, and subsequent bioactivity testing. The complete extraction and isolation workflow, including all fractionation and chromatographic steps, is illustrated in Figure 1.

The epiphytic biomass was pulverized and extracted with MeOH:CH_2_Cl_2_, followed by liquid–liquid partitioning between ethyl acetate (EA) and water (W). Preliminary TLC and ^1^H NMR analyses indicated that the aqueous phase was dominated by sugars and salts. Consequently, the EtOAc phase, which contained the majority of the non-polar to semi-polar metabolites, was selected for further investigation. This fraction was subjected to vacuum liquid chromatography (VLC), yielding a total of 13 subfractions (EA-1 to EA-13).

**Figure 2:**
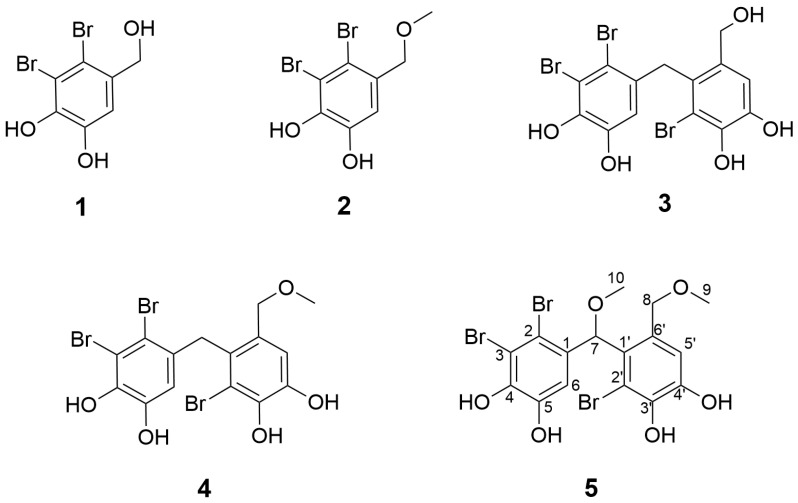
Bromophenols (**1**–**5**) isolated from the epiphytic biomass of *Laminaria hyperborea*. Compound **5** represents a new structure not previously reported in the literature.

All fractions were analyzed by analytical HPLC equipped with photodiode array (PDA) detection and by ^1^H NMR spectroscopy. Fractions EA-8, EA-9, and EA-10 contained the majority of brominated compounds, as indicated by UV absorption maxima (λmax ≈ 220 and 290 nm) characteristic of bromo-phenols (Figure S1), together with the presence of diagnostic bromine isotope abundance patterns in the corresponding mass spectra and signals in the aromatic region of the ^1^H-NMR spectra. Based on this combined UV–Vis, MS, and NMR-guided screening, EA-8 and EA-9 were further purified using silica gel chromatography (C18), Sephadex LH-20, and finally semi-preparative HPLC (see Section 4.3), resulting in the isolation of compounds **1**–**5**. The previously described bromophenols **1**–**4** were characterized by NMR (Table 1, Figures S2–S5) and high-resolution mass spectrometry (HR-MS) and confirmed to be identical to those reported in the literature [6,12,13] (Figure 2). Structural elucidation and identification of a new bromophenol, compound **5**, is given in Section 2.2.

### 2.2. Structural Elucidation of Compound ***5***

The previously unreported bromophenol **5** was isolated from the EA-9 fraction (Figure 1 and Figure 2). The compound was only partially pure and consisted of a mixture of the target bromophenol together with minor fatty acids.

It exhibited a molecular ion cluster consistent with a tribrominated molecule at *m*/*z* 538.8/540.8/542.8/544.8 (1:3:3:1), along with the corresponding [2M − H]^−^ cluster. The HR-ESI-MS at *m*/*z* 538.8336 [M − H]^−^ established the molecular formula C_16_H_15_Br_3_O_6_. The MS/MS spectra of **5** closely resembled that of **4**, showing no distinct fragmentation other than sequential loss of ^79^Br/^81^Br atoms and the neutral loss of 32 Da (CH_3_OH), typically associated with methoxy groups, and 28 Da (CO) (Figures S12–S14).

The ^1^H NMR spectrum displayed three singlets at *δ* 6.01 (1H, s, H-7), 6.47 (1H, s, H-6), and 7.08 (1H, s, H-5′) and two singlets belonging to methoxy groups at *δ* 3.34 (s, 3H, H-9) and 3.36 (s, 3H, H-10). Additionally, two doublets were observed at *δ* 4.45 (1H, d,12.5 Hz, H-8a) and 4.51 (1H, d,12.6 Hz, H-8b) that belong to the same AB spin system, confirmed by ^1^H-^1^H COSY correlations. The DEPT spectra showed 15 carbon signals comprising two methoxyls (C9, C10), two oxygenated sp^3^ carbons (C7, C8), and twelve sp^2^ carbons, two of them connected to H (C6, C5′), and four hydroxylated (two overlapping signals, C4 and C5, as seen by their chemical shifts around 145 ppm, C3′, C4′) (see Table 1). HSQC establishes all direct ^1^H–^13^C connectivities, enabling unambiguous assignment of the protonated carbons (Figures S6–S9).

All the above data support that **5** is another dibenzyl tribromophenol in which two benzyl units, 2,3-dibromo-4,5-dihydroxybenzyl and 2-bromo-3,4-dihydroxybenzyl, are linked through C-1/C-1′. The key structural differences between compound **5** and compounds **2** and **4** are the presence of an oxymethine bridge (C-7), replacing the methylene bridge, and the two diastereotopic protons of the benzylic methylene, which appear as two distinct signals rather than a singlet.

The HMBC spectrum provided key long-range correlations, H-7 to C-6, C-2, and C-1 within one aromatic ring, and H-7 to C-1′, C-2′, and C-6′ in the other, confirming the connectivity between the two moieties via the oxymethine bridge. Each methoxy group exhibited HMBC correlations exclusively to its attached methylene carbon, thereby excluding alternative placements (Figure S10).

**Figure 3:**
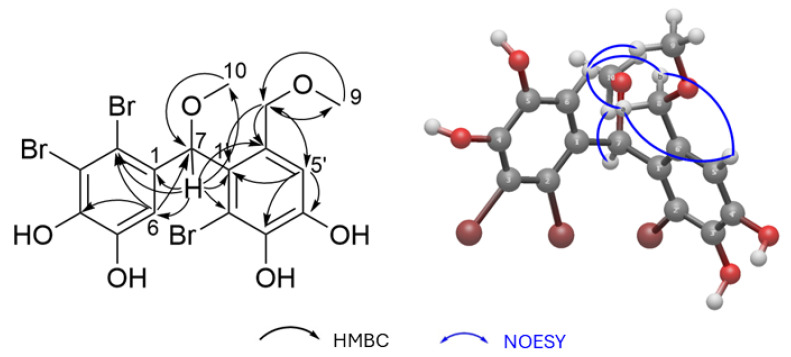
Key HMBC (black arrows) and NOESY (blue arrows) correlations for compound **5**, supporting the proposed connectivity and relative spatial arrangement of substituents.

The relative configuration of compound **5** was deduced from ^1^H–^1^H NOESY experiments. Strong correlations between H-6 and H-8a/H-8b, together with a weak one to H-9, indicate that H-6 is positioned on the same face as the alkyl chain of the opposite ring, with the bromine atoms oriented on the same side of the molecule. In contrast, the oxymethine proton H-7 and the methoxy protons H-10 showed strong mutual correlation only, placing them on the opposite face, away from the two aromatic planes (Figure S11). Key NOESY correlations and the proposed 3D configuration are illustrated in Figure 3.

Compound **5** contains a chiral center at C-7 and was found to be optically active ([α]^24^_D_: +18°, c 0.1, CHCl_3_). Accordingly, its structure was determined as (+)-2,2′,3-Tribromo-3′,4,4′,5-tetrahydroxy-6′-methoxy-methyl-diphenyl-methoxymethane. The absolute configuration (*R*/*S*) at C-7, however, remains to be assigned.

### 2.3. Cytotoxicity

**Figure 4:**
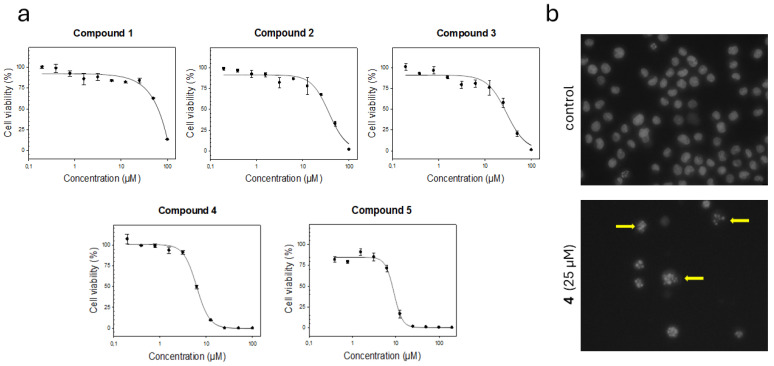
(**a**) Dose-response curve for compounds **1**–**5** against MOLM-13 leukemia cells after 48 h incubation. (**b**) Nuclear morphology of MOLM-13 cells. Cells were fixed and stained with the DNA dye Hoechst 33342 to visualize the nuclei. The upper image shows untreated cells, and the lower image shows cells treated with compound **4** at 25 μM concentration. The arrows indicate typical apoptotic nuclei. Images were acquired by fluorescence microscopy with a Nikon Diaphot 300 at 40× magnification.

To assess the anticancer potential of the four major isolated bromophenols (**1**–**4**), inhibitory effects on the proliferation of the acute myeloid leukemia (AML) cell line MOLM-13 were evaluated. Dose-dependent cell viability of MOLM-13 for compounds **1**–**4** is presented in Figure 4a. Compound **1** exhibited weak cytotoxicity, whereas compounds **2** and **3** showed moderate activity. In contrast, compound **4** demonstrated strong cytotoxic activity, with an EC_50_ value of 6.2 μM. Furthermore, compound **5** exhibited an EC_50_ value of approximately 9 μM. However, unlike compounds **1**–**4**, compound **5** was not obtained in pure form; therefore, the reported EC_50_ is approximate and not directly comparable to the EC_50_ values of the other compounds. The corresponding EC_50_ values are summarized in Table 2.

To determine whether the reduction in metabolic activity observed for compound **4** was caused by cell death and not a reduction in proliferation, the nuclear morphology of MOLM-13 cells was examined. Microscopic images of Hoechst-33342-stained nuclei revealed characteristic apoptotic features, such as chromatin hypercondensation and nuclear fragmentation (Figure 4b). Although further tests, like detection of cleaved caspase 3 and 7, or the TUNEL assay, would provide more evidence, the observed morphological changes strongly indicate that compound **4** induces cell death through apoptotic signals.

Compounds were also tested against the normal cell lines NRK (kidney) and H9c2 (cardiomyoblasts) to evaluate potential toxicity toward non-cancerous cells in vitro. Compounds **1** and **2** showed no detectable toxicity in either cell line, even at the highest tested concentration (100 μM). Compound **3** did not cause 50% inhibition at any concentration, with cell viability of 58% for NRK and 57% for H9c2 at 100 μM. Similarly, compound **4** reduced H9c2 cell viability to 53% only at 100 μM concentration. For NRK cells, compound **4** showed moderate toxicity, reducing the cell viability to 20% at 100 μM, with an estimated EC_50_ of approximately 25 μM. Compound 5 was found to be more toxic to both NRK and H9c2 than toward the MOLM-13 cells, with EC_50_ values of approximately 3 μM and 1 μM, respectively.

Based on these data, selectivity indices (SIs) were calculated and are summarized in Table 2. Among the tested compounds, bromophenol **4** exhibited the most favorable selectivity profile, with SI values of 4.0 for NRK and 16.1 for H9c2, indicating a selectivity window toward leukemia cells. In contrast, compound **5** showed no selectivity toward cancer cells.

### 2.4. Toxicity Towards Zebrafish Larvae

**Figure 5:**
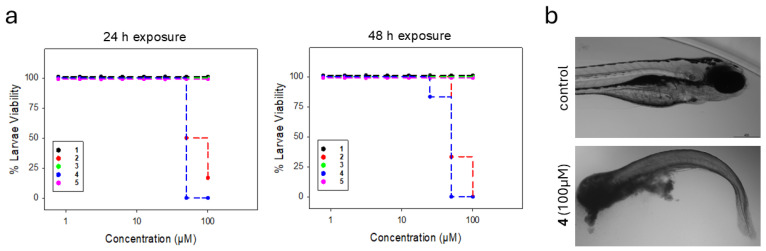
(**a**) Percentage viability of the zebrafish larvae incubated in different concentrations of compounds **1**–**5** after 24 h (**right**) and 48 h (**left**) of exposure, *n* = 6 larvae in each group; (**b**) Representative images of a zebrafish larva without and with treatment. Upper image (control) shows a healthy larva without treatment exhibiting no abnormalities; lower image shows larva exposed to 100 μM of compound **4** for 24 h exhibiting structural deformities and body breakdown.

The in vivo acute toxicity of the bromophenols was examined using a zebrafish larvae model. First, the lethality of the compounds dissolved in embryo water was determined. The larvae were exposed to concentrations ranging from 0.8 to 100 μM for 48 h. Compounds **1**, **3**, and **5** were well tolerated at all concentrations with no dead larvae observed after 48 h. However, exposure to compound **4** resulted in mortality at the highest concentrations, 100 and 50 μM, within the first 24 h, and for compound **2** at 100 and 50 μM after 48 h, while all lower concentrations were tolerated for the remaining duration (Figure 5a). The dead larvae exhibited necrosis with eventual rupture of the body (Figure 5b). In the control group, exposed only to decreasing concentrations of DMSO, all larvae survived for 48 h. Among the surviving larvae, no morphological abnormalities were detected.

### 2.5. Antioxidant Activity

The total antioxidant capacity of the isolated bromophenols was monitored by ABTS radical-scavenging assay. As summarized in Table 3, all compounds exhibited radical-scavenging ability to varying extents, but lower than the positive control of ascorbic acid. Among them, **4** demonstrated the highest activity with an EC_50_ of 22.1 μM, followed by **3** (34.2 μM), **2** (74.2 μM), and **1**, which showed weak activity.

### 2.6. Doxorubicin-Induced Toxicity

To examine if compound **4** could protect H9c2-cells from doxorubicin (Dox)-induced cell death, the H9c2 cells were exposed to increasing concentrations of Dox (0.25, 0.5, and 1 μM), together with compound **4** (12.5, 25, 50 μM), and incubated for 24 h. There was no significant change in cell survival between the cells incubated with Dox alone and those incubated with Dox together with compound **4** (Figure S15). It should be mentioned that cell viability was determined microscopically based on morphological assessment and counting rather than a metabolic assay (Section 4.7). Overall, it was concluded that **4** did not counteract Dox-induced cytotoxicity in cardiomyoblasts relative to Dox treatment alone.

### 2.7. LC-MS Characterization with Focus on Bromophenols

A comprehensive metabolite profile was obtained using non-targeted UHPLC-QTOF MS/MS analysis to identify more bromophenols in the epiphytic extract. The analysis was performed in the negative mode, as this provided optimal ionization for these compounds based on our previous experience. Several major chromatographic peaks (Figure S15) were identified as polybrominated compounds from their characteristic isotopic patterns, each containing one to three bromine atoms. In total, fourteen bromophenols were detected, including the five isolated (**1**–**5**), and nine tentatively identified based on their proposed molecular formulas, scoring metrics, and in-source fragmentation patterns (Table 4).

Among the bromophenols identified in this study, four were sulfated derivatives. Compound **7**, with [M − H]^−^ *m*/*z* 375/377/379, was tentatively characterized as a dibrominated phenol (C_7_H_6_O_6_SBr_2_) based on the 1:2:1 isotope and the formula prediction score. The MS/MS spectrum confirmed this assignment, showing an equally intense fragment cluster at *m*/*z* 295/297/ 299, typical for the lanosol scaffold (C_7_H_6_Br_2_O_3_). This fragment corresponds to the characteristic loss of an SO_3_ group (−80 Da) from the molecular ion via cleavage of the O-S bond, consistent with previous reports [14]. Additionally, a distinct fragment at *m*/*z* 96.9600 (≈97) was observed, attributed to HSO_4_^−^, produced by the C-O bond cleavage, further supporting the sulfate substitution [14,15]. Similar fragmentation was observed for compound **8**, which differed from compound **7** only by the presence of an additional bromine atom (1:2:2:1). Although lanosol and its tribrominated analog have been found in several red algae, sulfated lanosol has only been documented once, in the brown algae *Fucus vesiculosus* [16]. In general, sulfated compounds are prone to hydrolysis during extraction and isolation, which may lead to their underrepresentation in metabolite profiles [17].

Overall, in negative ion mode, the MS/MS spectra showed few fragment ions, primarily corresponding to losses of sulfate, methoxy, or bromine substituents. Because the fragmentation behavior of bromophenols has been rarely described in the literature, elucidating their fragmentation patterns and confidently identifying brominated phenolic residues remains challenging.

To determine the origin species of the bromophenols detected in the epiphytic biomass, the crude extracts of the major algal species were analyzed individually by LC-MS/MS. From the major species tested (Table S1), the two *Rhodomela* species contained several brominated metabolites, whereas the other prominent red algae—*Palmaria palmata, Ptilota gunneri*, and *Membranoptera alata*—lacked characteristic brominated isotope clusters. In *Rhodomela confervoides*, compounds **1**, **2**, **6**, and **9** were detected (Table 4); however, compounds **3**, **4**, and **10**, previously reported from this species, were not observed in the present sample, likely due to low abundance, masking by dominant metabolites, or environmental variation. On the contrary, *Rhodomela lycopodioides* contained nearly all bromophenols tentatively identified in the epiphytic extract, suggesting that it is the primary contributor to the bromophenol profile of the biomass.

## 3. Discussion

During the industrial processing of *Laminaria hyperborea*, the associated epiphytic biomass is routinely removed and discarded, despite being a biologically and chemically rich material. This biomass contains several red algae, which are potential reservoirs of structurally diverse metabolites and therefore represent a valuable yet largely underutilized source for bioactive compounds. To valorize this waste material, we focused on bromophenols, as they are among the most characteristic metabolites of Rhodophyta and exhibit a wide range of reported biological activities [2,4]. The crude extract of the epiphytes contained several bromophenols typical of the *Rhodomelaceae* family. However, due to the complexity of this biomass, multiple fractionation steps were required to target these compounds. Combined chromatographic techniques, ^1^H NMR, and MS analyses were employed to localize the major bromophenols to a few enriched fractions for isolation. This optimized workflow enabled the isolation of bromophenols in sufficient amounts, addressing the common challenge of low natural abundance and supporting their potential applicability for industrial use. Among the isolated metabolites, compounds **1**–**4** were the most abundant, while the first described compound **5** was present in minor amounts. In addition, nine additional bromophenols were tentatively identified by HRMS-QTOF.

Jacobtorweihen et al. categorized marine algae bromophenols into fifteen structural scaffold types to describe their phylogenetic distribution across taxa [18]. All compounds tentatively identified in this study fit within this classification: the 3-bromo-4-hydroxybenzyl type (A) (**6**, **9**), the lanosol type (B) (**1**, **2**, **7**, **10**, **11**), their dimeric forms (**3**, **4**, **5**, **13**, **14**), and the 2,3,6-tribromo-4,5-dihydroxybenzyl type (D) (**8**, **12**). Despite the abundance of sulfur in seawater and the plethora of sulfated marine compounds, only a few examples of sulfated bromophenols have been reported to date [15,19,20,21,22,23]. The sulfated compounds suggested in this study (**7**, **8**, **10**, **11**) share the aforementioned scaffolds, indicating that sulfation may occur as a secondary modification within these biosynthetic categories.

Overall, these findings align with previous reports on the chemical profile of the *Rhodomela* genus among red algae. The individual LC-MS/MS analyses of the dominant species strongly support that *R. lycopodioides* and, to a lesser extent, *R. confervoides* are the main contributors of bromophenols within the epiphytic biomass. *R. confervoides* is well established as one of the richest natural sources of bromophenols and has been reported to contain compounds **1**–**4** [5,6,22,24,25], along with several of the tentatively identified metabolites (**9**, **10**, and **11**) [5,15]. In contrast, the chemical profile of *R. lycopodioides* has not been previously described. To the best of our knowledge, this is the first study on the phytochemistry of this species, and our results highlight the importance of further investigating its bromophenolic diversity. Finally, our observations indicate that other dominant species of the epiphytic biomass, such as *Palmaria palmata*, *Ptilota gunneri*, and *Membranoptera alata,* do not synthesize bromophenols. It is worth mentioning that bromophenol content in marine algae is known to vary with season and location [9,26]. However, as the present material was collected at a single time point, temporal variability was not assessed and remains a subject for future investigation.

Although LC–MS is widely applied in marine metabolomics, bromophenols are rarely investigated using MS-based profiling approaches and are more commonly accessed through classical isolation workflows. The present results demonstrate that LC–MS-based profiling can facilitate mapping bromophenol diversity, tracing species origin, and guiding targeted isolation from complex biomasses, supporting more efficient and sustainable bioprospecting strategies.

To further explore the potential of the main bromophenols from epiphytes, the isolated compounds were evaluated for their cytotoxic, antioxidant, and cardioprotective activities. Bromophenols **1**–**5** were tested against leukemia cells, specifically acute myeloid leukemia (AML). When assessed on MOLM-13 cells, compound **4** exhibited the highest activity, with an EC_50_ value of 6.23 μM, notably lower than that of compound **3**, while compound **5** had a value of approximately 9 μM. To the best of our knowledge, this is the first report describing the cytotoxic activity of these bromophenols against leukemia cells. These results are consistent with previous reports on the cytotoxicity of compounds **1**–**4** against various cancer cell lines [27,28,29,30,31]. For instance, compound **4** has demonstrated activity against lung adenocarcinoma (A549) (19.5 μM), stomach cancer (BGC-823) (8.6 μM), hepatoma (Bel7402) (1.9 μM) [30], breast cancer (MCF-7) (17.6 μM), hepatoma (Bel7402) (1.9 μM), human malignant melanoma (B16-BL6) (15.5 μM), human sarcoma (HT-1080) (10.4 μM), and human colon cancer (HCT-8) (18.9 μM) [27].

Although these compounds exhibit activity across various cancer cells, their selectivity needs to be considered. Previous studies have not evaluated these bromophenols against any non-cancerous cell lines, representing a common limitation in natural product drug discovery. To address this gap, we assessed their toxicity toward two normal cell lines to determine selectivity. Compounds **1**–**3** showed no detectable cytotoxicity against either NRK or H9c2 cells. Compound **4**, consistent with its cytotoxic profile, exhibited increased toxicity in higher concentrations, particularly towards NRK cells, but maintained a tolerable selectivity window up to 25 μM.

While in vitro assays provide an initial indication of toxicity, they do not always reflect whole-organism responses. Zebrafish larvae are increasingly used as a predictive model, offering a more physiologically relevant assessment of drug safety [32]. In vivo observations in zebrafish larvae supported these interpretations; toxicity was limited to the highest concentrations of compounds **2** and **4**, whereas all lower doses were well tolerated and did not induce mortality or visible morphological abnormalities during the exposure period. In contrast, other bromophenols such as bis(2,3-dibromo-4,5-dihydroxybenzyl) ether (BDDE) have shown clearer signs of in vivo toxicity, including decreased hatching and increased mortality at similar concentrations [33].

Collectively, cellular and larval data indicate that compound **4** exhibits dose-dependent toxicity, occurring primarily at concentrations well above its effective range against leukemia cells. These findings provide a more comprehensive evaluation of overall toxicity and suggest promising anticancer potential for bromophenol **4** in terms of potency and selectivity.

In contrast, the new compound **5** displayed clear cytotoxic activity against MOLM-13, but, unlike compounds **1**–**4**, it was more toxic to the normal cells in vitro. However, when tested in vivo, no mortality or morphological changes were detected in zebrafish larvae at the tested concentrations. This reflects the difference between direct exposure to cultured cells and whole organisms, where factors such as uptake, distribution, and metabolism must be considered. It is possible that the larvae did not take up enough of compound **5**, or that any toxic effects were buffered or metabolized in vivo. As a result, the toxicity observed in cell assays does not necessarily translate into acute toxicity in the zebrafish model under the conditions tested. But overall, the lack of selectivity, together with the fact that the sample is partially pure, limits the interpretation of its biological potential. The observed toxicity toward normal cells may be influenced by non-selective cytotoxic effects of co-isolated fatty acids or other impurities. Therefore, the selectivity of the bromophenol itself remains uncertain.

In addition to cytotoxicity, compounds **1**–**4** were evaluated for antioxidant activity. As expected for phenolic structures and consistent with previous reports [5,25,31], these bromophenols acted as effective radical scavengers. Free radicals play a central role in the pathogenesis of several diseases, including cancer, and such activity may be related to the observed cytotoxic effects. Compound **4** again demonstrated the strongest ABTS radical-scavenging activity, with an IC_50_ value of 22.1 μM, while **1**–**3** had moderate activity. Although overall ABTS activity was lower than that of the positive control (ascorbic acid), contradicting previous findings by Li et al., the relative potency trend (**4** > **3** > **2** > **1**) was consistent with earlier studies [5,25].

Compounds with radical-scavenging activity could potentially protect cardiomyocytes against Dox-induced cytotoxicity. Dox is a widely used anti-cancer drug, but it is associated with severe side effects like cardiomyopathy, which can lead to heart failure. One mechanism underlying this toxicity is the generation of reactive oxygen species (ROS) during its intracellular metabolism, which triggers oxidative stress pathways leading to mitochondrial dysfunction and cellular damage [34]. Considering the central role of oxidative stress in this pathophysiology and the antioxidant capacity of compound **4**, we co-administered it with Dox to reduce oxidative damage in heart cells. The results showed no significant improvement in cell viability. However, this outcome may not fully represent cardiotoxic events in vivo. Previous studies have demonstrated that short-term reduction in ROS levels does not necessarily correlate with decreased cytotoxicity in vitro [35]. Nevertheless, the possibility that the antioxidant capacity of compound **4** is insufficient to counteract ROS accumulation cannot be excluded.

The cytotoxicity against MOLM-13 cells and antioxidant results (Table 1) indicate that activity is strongly influenced by the number and position of hydroxyl and bromine substituents. In both assays, dimeric compounds exhibited higher activity than monomeric analogues. Consistent with previous reports, the 2,3-dibromo-4,5-dihydroxybenzyl scaffold appears to be a key structural determinant for bioactivity [11]. It has been found that extensive hydroxyl substitution, particularly ortho-dihydroxyl substitution, enhances the antioxidant activity of bromophenols in radical-scavenging assays [36]. This trend is reflected in our results, where dimeric compounds **3** and **4**, bearing more hydroxyl groups, showed superior radical-scavenging capacity. Additional studies suggest that hydroxylation generally plays a more significant role than bromination in the antioxidant activity [37], whereas bromine substituents appear to have a stronger influence on cytotoxic effects [28].

The methylation of hydroxyl groups also affected the activity. The most active compound in each series was the methylated derivative: bromophenol **2** is more active than **1**, and **4** is more active than **3**. Typically, methylation of phenolic hydroxyl groups reduces antioxidant activity because methylated hydroxyls cannot donate hydrogen atoms and provide weaker electron-donating effects, resulting in less stabilized phenoxy radicals [38,39]. Similarly, methylation often diminishes cytotoxicity [28]. However, methylation of a hydroxyl group on a side chain rather than directly on the aromatic ring does not interfere with the aromatic O–H bond and therefore does not reduce radical-scavenging activity. On the contrary, the alkyl substituents have an important role in bioactivity, and elongation of the side chain appears to enhance both the antioxidant and the cytotoxic capacity, as reported previously [5,25,28] and confirmed in this study. This effect may be explained by favorable changes in molecular conformation, improving the accessibility of aromatic hydroxyl groups for hydrogen donation to free radicals. Increased lipophilicity due to methylation may also enhance membrane permeability and interactions with intracellular targets. Furthermore, conformational changes due to methylation could improve binding to proteins or enzymes involved in cytotoxic mechanisms. Although these interpretations remain hypothetical, they align with previous findings that even minor changes in substitution patterns can significantly influence target affinity and, consequently, both antioxidant and cytotoxic profiles of bromophenols. These observations provide a preliminary structure–activity relationship that may guide the synthesis of analogues and deepen understanding of their underlying mechanisms, which should be addressed in future mechanistic and in vivo studies.

## 4. Materials and Methods

### 4.1. Biological Material

The epiphyte biomass was acquired from Alginor ASA (Haugesund, Norway). *Laminaria hyperborea*, with its epiphytes, was harvested from the coast of Haugesund, Norway, field 56A (59°12′26.6″ N, 5°09′14.2″ E), in November 2022. The epiphyte biomass had been collected through gentle mechanical scraping and frozen immediately after separation. After receiving the biomass, the collected epiphyte material was washed thoroughly with water, and the species were separated and examined macroscopically and microscopically, as described previously [8] (Table S1). Next, 1.5 kg of frozen biomass was freeze-dried, milled with a kitchen-type blender, and stored at −20 °C when not in use.

### 4.2. Instruments and Chemicals

All solvents used were of analytical and HPLC grade from Sigma-Aldrich Inc. (Sigma-Aldrich, St. Louis, MO, USA). Ultrapure water was either deionized at the University of Bergen or produced by a Millipore Milli-Q Direct 8 water purification system. Stationary phases for column chromatography were Silica gel 60, 230–400 mesh (Merck KGaA, Darmstadt, Germany), Sephadex LH-20 (Cytiva, Marlborough, MA, USA), XAD-7 (Sigma-Aldrich), and C18-Silica gel (Merck KGaA).

All NMR spectra (^1^H, ^13^C, and 2D) were recorded in methanol-d4 on a Bruker 600 MHz and 850 MHz AVANCE NEO 850 MHz instrument (Bruker BioSpin, Zürich, Switzerland) at 25 °C. Chemical shifts are expressed in δ (ppm), and the following abbreviations were used to describe the multiplicities: s = singlet, d = doublet.

High-resolution mass spectrometry ESI MS data were measured on an Agilent QTOF (Agilent Technologies, Santa Clara, CA, USA) coupled with an Agilent Technologies 1260 Infinity Series system. ESI operation conditions: 12 L/min N_2_, 310 °C drying gas temperature, 35 psig nebulizer, and 4000 V capillary in negative ionization mode. DDA data were recorded in Auto MS/MS mode; full scan and MS/MS spectra were acquired within a range of 60 to 1500 *m*/*z*, 10, 20, 30, and 40 eV collision energy A Luna Omega C18 (Phenomenex, Torrance, CA, USA) UHPLC column 150 × 2.1 mm, 1.6 μm was used for separation with a gradient of 5 to 25% B for 5 min, 25 to 100% B for 10 min, and 100% B for 10 min at a 0.350 mL/min flow rate, where A is water with formic acid 0.1% *v*/*v* and B is acetonitrile (MeCN) with formic acid 0.1% *v*/*v*. UV–vis spectral data for all peaks were obtained in the range of 200–800 nm and at 210, 220, 280, and 290 nm.

Optical rotation was measured using an Autopol IV (Rudolph Research Analytical, Hackettstown, NJ, USA) at 24 °C and 589 nm. The compound concentration was 0.1 g/100 mL, and the solvent used was CH_3_Cl.

For the cell assays, DMEM (D6429) and RPMI medium (R5886), penicillin/streptomycin (P0781), glutamine (G7513), fetal bovine serum (F7524), formaldehyde, and phosphate-buffered saline tablets (PBS) were purchased from Sigma-Aldrich (St. Louis, MO, USA). Cell Counting Kit-8 (CCK-8) was purchased from MedChemExpress (Monmouth Junction, NJ, USA), and Hoechst 33342 fluorescent DNA staining reagent from Thermo Fisher Scientific (Waltham, MA, USA).

### 4.3. Extraction and Isolation

The dried epiphytic biomass (450 g) was extracted with methanol (MeOH) and dichloromethane (DCM) with an ultrasonic bath at room temperature. The MeOH/DCM extract was evaporated to dryness, resulting in an oily dark green residual (185 g). The crude was suspended and partitioned successively with EtOAc (EA) and water (WP). The EA fraction (12 g) was subject to vacuum column chromatography (VCC) in a stepwise elution with n-hexane: acetone (100:1 to 2:1) followed by DCM:MeOH (20:1 to 0:100), each 300 mL, yielding a total of 13 fractions (EA-1 to EA-13). The EA-9 fraction (440 mg) eluted with 20:1 DCM:MeOH was chromatographed over reversed-phase silica gel chromatography (C18 CC), eluting with a gradient increasing acetone (20–100%) in water, and finally flushed by DCM and separated into 6 main subfractions (EA-9-1 to EA-9-6). Subfraction EA-9-3 (90 mg), eluted with 60% acetone, was then subjected to preparative HPLC (Ascentis C18, 250 × 21.2 mm, 5 μm) with a gradient of 35 to 100% B in A for 30 min at a 10 mL/min flow rate, where A is water with formic acid 0.1% *v*/*v* and B is acetonitrile (MeCN) with formic acid 0.1% *v*/*v*. UV was set to 220 and 280 nm. This yielded the four main compounds **2** (10 mg), **3** (17 mg), **4** (27 mg), and **5** (1.5 mg). To obtain a larger quantity of the above, the EA-8 fraction (270 mg) was chromatographed over Sephadex LH-20, eluting with DCM:MeOH, to give six subfractions (EA-8-1 to EA-8-6). Subfraction EA-8-5 and EA-8-6 were subject to semi-preparative HPLC (Luna C18, 250 × 10 mm, 5 μm) with a gradient of 0 to 20% B for 5 min and 20 to 100% B for 30 min at a 4.5 mL/min flow rate, where A is water with formic acid 0.1% *v*/*v* and B is acetonitrile (MeCN) with formic acid 0.1% *v*/*v*. UV was set to 220 and 280 nm. This yielded more of compounds **2**–**4**, as well as small amounts of compound **1** (2 mg). All the separation steps were monitored on the basis of TLC, HPLC, and NMR analyses. Their chemical structures were determined based on NMR spectral data and HRMS data and compared with the literature.

*Lanosol, 2,3-dibromo-4,5-dihydroxybenzyl alcohol, (3,4-dibromo-5-(hydroxymethyl)benzene-1,2-diol) **(1)***: brown–pink solid; UV (MeCN, water) *λ*max 214, 292 nm; ^1^H and ^13^C NMR data Table 1; HRESI-MS *m*/*z* 294.8609 [M − H]^−^ (calcd. for C_7_H_5_^79^Br_2_O_3_^−^, 294.8611); observed adducts and fragments: 408.8534 [M + TFA − H]^−^, 374.7860 [M + Br]^−^, 590.7289 [2M − H]^−^, 214.9347 [M − HBr − H]^−^.

*Lanosol methyl ether, 2,3-dibromo-4,5-dihydroxybenzyl methyl ether, (3,4-dibromo-5-(methoxymethyl)benzene-1,2-diol) **(2)***: brown–pink solid; UV (MeCN, water) *λ*max 214, 292 nm; ^1^H and ^13^C NMR data, Table 1; HRESI-MS *m*/*z* 308.8766 [M − H]^−^ (calcd. for C_8_H_7_^79^Br_2_O_3_^−^, 308.8767); observed adducts and fragments MS1: 422.8689 [M + TFA − H]^−^, 388.8021 [M + Br]^−^.

*2,2′,3-tribromo-3,4,4′,5-tetrahydroxy-6′-hydroxymethyldiphenylmethane, (5-(2-bromo-3,4-dihydroxy-6-(hydroxymethyl)benzyl)-3,4-dibromobenzene-1,2-diol) **(3)***: bright pink solid; UV (MeCN, water) *λ*max 220, 290 nm; ^1^H and ^13^C NMR data, Table 1; HRESI-MS *m*/*z* 494.8073 [M − H]^−^ (calcd. for C_14_H_10_^79^Br_3_O_5_^−^, 494.8084); observed adducts and fragments MS1: 608.7998 [M + TFA − H]^−^, 574.7345 [M + Br]^−^, 990.6241 [2M − H]^−^.

*2,2′,3-tribromo-3′,4,4′,5-tetrahydroxy-6′-methoxymethyldiphenylmethane, (5-(2-bromo-3,4-dihydroxy-6-(methoxymethyl)benzyl)-3,4-dibromobenzene-1,2-diol) **(4)***: pink–brown solid; UV (MeCN, water) *λ*max 236, 290 nm; ^1^H and ^13^C NMR data, Table 1; HRESI-MS *m*/*z* 508.8230 [M − H]^−^ (calcd. for C_15_H_13_^79^Br_3_O_5_^−^, 508.8240); observed adducts and fragments MS1: 622.8157 [M + TFA − H]^−^, 588.7490 [M + Br]^−^, 1018.6530 [2M − H]^−^.

*2,2′,3-tribromo-3′,4,4′,5-tetrahydroxy-6′-methoxymethyldiphenyl-methoxymethane, (5-((2-bromo-3,4-dihydroxy-6-(methoxymethyl)phenyl)(methoxy)methyl)-3,4-dibromobenzene-1,2-diol) **(5)***: pale pink oil; [α]^24^_D_ = +18° (c 0.1, CHCl_3_); UV (MeCN, water) *λ*max 218, 290 nm; ^1^H and ^13^C NMR data, Table 1; HRESI-MS *m*/*z* 538.8336 [M − H]^−^ (calcd. for C_16_H_14_^79^Br_3_O_6_^−^, 538.8346); observed ion cluster at *m*/*z* 538.8336, 540.8320, 542.8301, and 544.8283 with relative intensities 1.06:3.09:3.01:1.00; adducts and fragments MS1: 652.8264 [M + TFA − H]^−^, 618.7540 [M + Br]^−^, 1078.6733 [2M − H]^−^, 458.9077 [M − HBr − H]^−^, 508.8070 [M − MeOH − H]^−^, 426.8819 [M − MeOH − HBr − H]^−^; main fragments MS/MS: 474.7823 [M − 2MeOH − H]^−^, 395.8638 [M -2 MeOH − Br − H]^−^, 314.9300 [M − 2MeOH − 2Br − H]^−^, see Figure S14 for fragmentation.

### 4.4. Sample Preparation for LC-MS

The algal materials *Palmaria palmata, Ptilota gunneri, Membranoptera alata, Rhodomela lycopodioides*, and *Rhodomela confervoides* were separated from the epiphytic biomass, cleaned, freeze-dried, milled, and extracted with MeOH, as in Section 4.3, to obtain the crude extracts. A few mg of each extract, as well as the epiphytic biomass extract, were re-dissolved in MeOH to a concentration of approximately 2 mg/mL. The samples were qualitatively analyzed with an Agilent QTOF LC-MS system, as described in Section 4.2.

### 4.5. ABTS Antioxidant Assay

The ABTS method by Re et al. [40] was slightly modified and optimized for use with a microplate spectrophotometer. An ABTS stock solution was prepared from a mixture 1:1 of a 7 mM ABTS solution and a 2.45 mM K_2_S_2_O_8_ solution, both in distilled water, and was incubated in the dark for at least 12 h. The stock solution was used within 5 days after preparation. The ABTS working solution was prepared from a dilution of 1:10 of the stock solution with water. A 20 µL sample in dimethyl sulfoxide (DMSO) was mixed with 80 µL distilled water and 180 µL of the working solution and then incubated in the dark for 30 min. Absorbance was measured at 734 nm. Ascorbic acid was used as a positive control for comparison.

The EC_50_ was calculated from a four-parameter non-linear regression analysis model of the percentage decrease in the absorbance of the ABTS solution versus the concentration of the sample. All experiments were performed in triplicate (duplicate for compound **2**), and mean values were calculated for each treatment ± standard deviation. All statistical analyses were carried out using SigmaPlot.

### 4.6. In Vitro Cytotoxicity

#### 4.6.1. Cell Cultures

Compounds **1**–**5** were tested for their activity against human acute myeloid leukemia (AML) cell line MOLM-13 (DSMZ, ACC-554) [41] and the non-cancerous cell lines, normal epithelial rat kidney cell line NRK (ATCC, CRL-6509), and normal rat cardiomyoblast line H9c2 (ATCC, CRL-1446). MOLM-13 cells were cultured in RPMI medium, and NRK and H9c2 were cultured in Dulbecco’s modified Eagle’s medium (DMEM), both supplemented with 10% (*v*/*v*) fetal serum (FBS), 0.2 mM L-glutamine, 100 IU/mL penicillin, and 0.1 mg/L streptomycin. The MOLM-13 cells were suspended and cultured to a density of 10–80 × 10^4^ cells/mL and diluted by adding fresh medium. The adherent cell lines H9c2 and NRK were cultured until they reached 80–90% confluence. They were then detached by mild trypsinization, centrifuged at 200× *g* for 5 min, and reseeded in fresh medium at around 30% and 10% confluence, respectively. Cells were incubated at 37 °C in a humidified atmosphere with 5% CO_2_. Adherent cells that had undergone more than 14 passages were not used.

#### 4.6.2. Cell Viability Assay

For cytotoxic testing, MOLM-13 cells were seeded at 40,000 cells/well in 96-well microplates with 100 mL medium/well on the day of the experiment. The adherent cells were seeded the day before the experiment to allow the cells to attach to the substratum. H9c2 cells were seeded at 3000 cells/well, and NRK cells were seeded at 2000 cells/well in 96-well microplates with 100 mL medium/well. The cells were exposed to various concentrations of the compounds (dissolved in DMSO) for 48 h and incubated in the same conditions as described in Section 2.6; 1% DMSO was used for comparison. The cell viability was assessed by CCK-8 reagent, following the manufacturer’s instructions. The assay is based on the ability of the mitochondria of the viable cells to convert the CCK-8 reagent into a soluble formazan dye, which can be measured spectrophotometrically, and the degree of conversion is proportional to the number of viable cells. The plates were further incubated for 2 h before the signal was recorded at 450 nm with reference at 620 nm using a Multilabel reader. Percent growth inhibition of cells exposed to treatments was calculated as follows:

(1) $$\%\;survival=\frac{\left(A-Ab\right)}{\left(Ac-Ab\right)}\;\times \;100$$

where A is the absorbance of the treated well with the sample; A_c_ is the absorbance of the control; A_b_ is the absorbance of the blank medium. Four-parameter non-linear regression analysis was used to calculate EC_50_ values from the CCK-8 assay data. Data analysis was performed using Office Excel 365 (Microsoft) and SigmaPlot 16.0 (Grafiti).

Selectivity indices (SIs) were calculated according to the following equation:

(2) $$SI=\frac{EC50\;normal\;cells}{EC50\;cancer\;cells}$$

using the mean EC_50_ values. For normal cell lines NRK and H9c2, where EC_50_ values exceeded the highest tested concentration, SI values are reported as minimum estimates.

The cells were next fixed by adding 100 mL 4% buffered formaldehyde (pH 7.4) containing 0.01 mg/mL of the DNA-specific fluorescent dye, Hoechst 33342, and the morphology of the nuclei was visualized by fluorescence microscopy using a Nikon Diaphot 300 microscope fitted with a 40 × Flu-Phase contrast lens and a DS-Fi3 camera.

All experiments were performed in triplicate (quadruplicate for compound **4**), and mean viability values were calculated for each treatment ± standard deviation. All statistical analyses were carried out using SigmaPlot. One-way ANOVA and Holm–Sidak tests with multiple comparisons, α = 0.05, were performed. Values of *p* < 0.05 were considered significantly different.

### 4.7. In Vivo Testing in Zebrafish Larvae

#### 4.7.1. Zebrafish Larvae Handling

Fertilized zebrafish (*Danio rerio*) eggs of the AB (ZFIN ID: SDB-GENO-960809-7) strain were obtained from the Zebrafish Facility at the Department of Biological Sciences, University of Bergen. The facility is run according to the European Convention for the Protection of Vertebrate Animals used for Experimental and Other Scientific Purposes. The embryos were kept in petri dishes with E3 medium containing 4.5 mM NaCl, 0.15 mM KCl, 0.30 mM CaCl_2_, and 0.30 mM MgSO_4_ in ddH_2_O at 28.5 °C, and dead eggs were removed during the first 24 h post fertilization. At day 5 post-fertilization, the larvae were euthanized by cooling on ice for 60 min before being transferred to −20 °C.

#### 4.7.2. Toxicity in Zebrafish Larvae

One zebrafish larva was added to each well in a 96-well plate containing a total of 100 μL of embryo water containing the compounds at different concentrations. Compounds **1**–**5** and control DMSO (starting concentrations, 1% *v*/*v*) were tested in six parallels in dilution series. The plate with the larvae was incubated in the dark at 28.5 °C and studied after 24 and 48 h for signs of toxicity. Larvae were examined under a Nikon Diaphot 300 microscope fitted with a 10× Flu-Phase contrast lens and a DS-Fi3 camera.

### 4.8. Dox-Induced Toxicity Test

For the doxorubicin-induced toxicity test, the H9c2 cells were seeded at 5000 cells/well in 96-well microplates with 100 mL medium/well one day before adding the drugs. During the experiment, the cells were exposed to various concentrations of Dox, 4, 2, 1, 0.5, and 0.25 μM, incubated for 1 h, and then the bromophenols were added at concentrations of 12.5, 25, and 50 μM for 24 h.

Due to the interference of Dox with the CCK-8, cell viability was assessed by fluorescence microscopy based on morphological evaluation and manual counting of live and dead cells. For each well of interest, multiple random microscopic fields were captured, with a minimum of 100 cells for each well, and the percentage of viable cells was calculated relative to the total number of cells observed. All experiments were performed in duplicate, and mean viability values were calculated for each treatment ± standard deviation. All statistical analyses were carried out using SigmaPlot. Student’s *t*-test, α = 0.05, was performed to compare each concentration of the drug to the DOX-alone control treatment. Values of *p* < 0.05 were considered significantly different.

## 5. Conclusions

This study demonstrates that epiphytic biomass from *Laminaria hyperborea* represents a valuable source of bromophenols, including one previously undescribed compound (**5**). The isolated bromophenols exhibited notable cytotoxicity against AML cells, with compound **4** showing the highest potency and a favorable selectivity supported by in vitro and in vivo zebrafish assays. In contrast, although compound **5** displayed cytotoxicity, its limited purity and lack of in vitro selectivity hinder the conclusions about its biological potential. Additionally, these compounds exhibited antioxidant activity, and preliminary structure–activity relationships suggest that hydroxylation, bromination, and methylation patterns significantly influence bioactivity. Finally, non-targeted LC–MS/MS proved useful for characterizing bromophenol diversity within a complex mixture, revealing nine additional bromophenols and indicating that *Rhodomela lycopodioides* and *R. confervoides* are the primary contributors to the profile of the biomass. These findings highlight the potential of epiphytic waste as a sustainable resource for bioactive compounds and provide a foundation for future studies on the isolation and pharmacology of bromophenol-based therapeutics.

## Figures and Tables

**Table 1:** The ^1^H and ^13^C NMR spectroscopy data for compounds **1**–**5** ^a^.

No	1		2		3		4		5	
	*δ*_H_ ^b^	*δ*_C_ ^c^	*δ*_H_ ^b^	*δ*_C_ ^c^	*δ*_H_ ^b^	*δ*_C_ ^c^	*δ*_H_ ^b^	*δ*_C_ ^c^	δ_H_ ^b^	δ_C_ ^c^
1		134.0		131.0		132.6		132.79		132.2
2		114.2		115.3		116.3		116.2		118.6
3		114.0		114.3		114.2		114.2		115.2
4		144.9		145.3		143.9		143.9		145.6 ^d^
5		146.2		146.3		146.3		146.2		145.6 ^d^
6	7.01, s, 1H	114.9	6.92, s, 1H	115.8	6.05, s, 1H	115.1	6.07, s, 1H	115.1	6.47, s, 1H	116.1
1′						129.0		130.3		127.1
2′						115.5		115.7		114.5
3′						143.8		144.4		143.7
4′						145.9		145.7		146.7
5′					6.96, s, 1H	115.6	6.87, s, 1H	116.8	7.08, s, 1H	115.7
6′						133.5		130.3		131.8
7	4.56, s, 2H	65.7	4.43, s, 2H	75.9	4.13, s, 2H	39.7	4.12, s, 2H	39.8	6.01, s, 1H	87.2
8			3.40, s, 3H	58.5	4.36, s, 2H	63.2	4.19, s, 2H	74.1	4.42, d, 1H, 12.6 Hz	73.3
									4.51, d, 1H, 12.5 Hz	
9							3.28, s, 3H	58.3	3.34, s, 3H	58.6
10									3.36, s, 3H	57.8

^a^ All NMR data were measured in *d*_4_-methanol. ^b^ Recorded at 850 MHz. ^c 13^C chemical shifts were assigned from ^13^C NMR, recorded at 214 MHz, DEPT, and HMBC spectra. ^d^ C-4 and C-5 could not be differentiated based on available evidence.

**Table 2:** Cytotoxic activity of compounds **1**–**5** against the MOLM-13 cell line.

Compound	EC_50_ (μM)			SI	
	MOLM-13	NRK	H9c2	NRK	H9c2
**1**	60.3 ± 5.8 ^a^	>100	>100	>1.7	>1.7
**2**	47.7 ± 3.8 ^b^	>100	>100	>2.1	>2.1
**3**	38.8 ± 3.5 ^c^	>100	≥100	>2.6	≥2.6
**4**	6.2 ± 0.2 ^d^	≥25	≥100	≥4.0	≥16.1
**5**	≈9 *	≈3 *	≈1 *	n.a.	n.a.

Results are presented as mean EC_50_ values obtained from three independent experiments (four for compound **4**) ± S.D; distinct superscript lowercase letters (a to d) denote significant differences among compounds. * The EC_50_ value is approximate and calculated based on the molar concentration of the bromophenol and does not account for the presence of fatty acids. SI represents the selectivity index; n.a., not applicable.

**Table 3:** ABTS radical-scavenging activity of compounds **1**–**4**.

Compound	EC_50_ (μM)
**1**	279.2 ± 38.7
**2**	74.2 ± 3.3
**3**	34.2 ± 1.9
**4**	22.1 ± 0.8
Ascorbic acid	5.1 ± 0.1

Results are presented as mean EC_50_ values obtained from three independent experiments (two for compound **2**) ± S.D.

**Table 4:** Tentatively identified bromophenols in epiphytes crude extract using UHPLC-HRMS.

	t_R_ (min)	*m*/*z* [M − H]^−^	*m*/*z* Calc [M − H]^−^	Molecular Formula	Error (ppm)	Score	Fragments	Suggested Compound	Rhodomela Confervoides	Rhodomela Lycopodioides
1	7.55	294.8606	294.8611	C_7_H_6_Br_2_O_3_	−1.70	92.6	295/297/299, 79/81, 107	**1**	*	*
2	9.43	308.8764	308.8767	C_8_H_8_O_3_Br_2_	−0.97	98.0	309/311/313, 277/279/281, 79/81	**2**	*	*
3	9.49	494.8079	494.8084	C_14_H_11_O_5_Br_3_	−1.01	92.5	495/497/499/501, 397/399/401, 79/81, 209, 289/291, 237, 153	**3**	n.d.	*
4	10.56	508.8231	508.8240	C_15_H_13_O_5_Br_3_	−1.77	97.9	509/511/513/515, 397/399/401, 79/81, 317/319, 289/291, 209, 237, 153	**4**	n.d.	*
5	10.35 ^a^	538.8341	538.8346	C_16_H_15_O_6_Br_3_	−0.93	94.8	539/541/543/545, 396/398/400, 475/477/479/481, 427/429/431, 79/81, 314/316/318, 236, 289/291, 209	**5**	n.d.	*
6	5.02 ^a^	216.9504	216.9506	C_7_H_7_O_3_Br	−0.92	98.6	217/219, 79/81, 200/202, 137, 165/167	Monobromophenol	*	*
7	7.22	374.8181	374.8179	C_7_H_6_O_6_SBr_2_	0.53	95.9	375/377/379, 295/297/ 299, 97, 79/81, 107	Dibromophenol sulfate	n.d.	*
8	7.60	452.7275	452.7284	C_7_H_5_O_6_SBr_3_	−1.99	96.6	453/455/457/459, 373/375/377/379, 97, 79/81, 185/187	Tribromophenol sulfate	n.d.	*
9	7.90	230.9661	230.9662	C_8_H_9_O_3_Br	−0.43	93.8	231/233, 79/81, 199/201, 133/135	Monobromophenol	*	*
10	8.84	388.8335	388.8336	C_8_H_8_O_6_SBr_2_	−0.26	95.7	389/391/393, 309/311/313, 111, 79/81, 197/199, 277/279/281	Dibromophenol sulfate	n.d.	*
11	9.27	292.8449	292,8454	C_7_H_4_O_3_Br_2_	−1.71	96.6	293/295/297, 79/81	Lanosol type	n.d.	*
12	10.02	466.7431	466.7441	C_8_H_7_O_6_SBr_3_	−2.14	97.5	467/469/471/473, 387/389/391/393, 111, 79/81, 275/277/279	Tribromophenol sulfate	n.d.	*
13	10.46 ^a^	492.7922	492.7927	C_14_H_9_O_5_Br_3_	−1.01	98.1	493/495/497/499, 413/415/417, 333/335, 79/81, 384/386/388, 225, 197	Dibenzyl tribromophenol	n.d.	*
14	11.02 ^b^	506.8083	506.8084	C_15_H_11_O_5_Br_3_	−0.20	89.6	507/509/511/513, 382/384/386, 427/429/431, 79/81, 302/304 209	Dibenzyl tribromophenol	n.d.	*

^a^ Observed in fraction EA-9, not crude. ^b^ Observed in fraction EA-9-3, not crude. * Present in sample; n.d. = not detected.

## Data Availability

All data included in this work are available upon request.

## Supplementary Materials

The following supporting information can be downloaded at: https://www.mdpi.com/article/10.3390/md24010052/s1. Figure S1: UV for compounds **1**–**5**; Figures S2–S11: Supporting NMR spectra for compounds **1**–**5**; Figures S12–S14: Supporting MS and MS/MS spectra for compounds **1**–**5**; Figure S15: Cytoprotective activity of compound **5** against Dox-induced cytotoxicity in H9c2 cells; Figure S16: Extracted ion chromatogram of major bromophenols in the crude extract of the epiphytes; Table S1. List of detected species and their relative contribution to epiphytic biomass.
