# Supplementary material for: Marine Bromophenols from Laminaria hyperborea’s Epiphytic Biomass: Chemical Profiling, Cytotoxicity, and Antioxidant Activity
Source: Mar Drugs. 2026 Jan 21;24(1):52. doi: 10.3390/md24010052 (PMC12843181; doi:10.3390/md24010052)
Supplement: Supplementary file 1 [file marinedrugs-24-00052-s001.zip › marinedrugs-4067549-supplementary.pdf]

marine drugs

MDPI

*Article*

# Marine Bromophenols from *Laminaria hyperborea* Epiphytes: Chemical Profiling, Cytotoxic and Antioxidant Activity

**Angeliki Barouti [1], Vinh Le Ba [1], Lars Herfindal [2] and Monica Jordheim [1*]**

1 Department of Chemistry, Faculty of Natural Sciences and Technology, University of Bergen, Bergen, Norway; angeliki.barouti@uib.no; vinh.ba@uib.no
2 Centre for Pharmacy, Department of Clinical Science, Faculty of Medicine, University of Bergen, Bergen, Norway; e-mail@e-mail.com
* Correspondence:

# Supporting Information

## Table of Contents:

**Figure S1**. UV spectrum of compounds **1–4** in methanol
**Figure S2**. $^1$H NMR spectrum of *2,3-dibromo-4,5-dihydroxybenzyl alcohol* (lanosol) (**1**) in $CD_3OD$
**Figure S3**. $^1$H NMR spectrum of *2,3-dibromo-4,5-dihydroxybenzyl methyl ether* (lanosol methyl ether) (**2**) in $CD_3OD$
**Figure S4**. $^1$H NMR spectrum of *2,2′,3-tribromo-3,4,4′,5-tetrahydroxy-6′-hydroxymethyldiphenylmethane* (**3**) in $CD_3OD$
**Figure S5**. $^1$H NMR spectrum of *2,2′,3-tribromo-3′,4,4′,5-tetrahydroxy-6′-methoxymethyldiphenylmethane* (**4**) in $CD_3OD$
**Figure S6**. $^1$H NMR spectrum of *2,2′,3-tribromo-3′,4,4′,5-tetrahydroxy-6′-methoxymethyldiphenyl-methoxymethane* (**5**) in $CD_3OD$
**Figure S7**. DEPT spectrum of *2,2′,3-tribromo-3′,4,4′,5-tetrahydroxy-6′-methoxymethyldiphenyl-methoxymethane* (**5**) in $CD_3OD$
**Figure S8**. $^1$H-$^1$H COSY spectrum of *2,2′,3-tribromo-3′,4,4′,5-tetrahydroxy-6′-methoxymethyldiphenyl-methoxymethane* (**5**) in $CD_3OD$
**Figure S9**. HSQC spectrum of *2,2′,3-tribromo-3′,4,4′,5-tetrahydroxy-6′-methoxymethyldiphenyl-methoxymethane* (**5**) in $CD_3OD$
**Figure S10**. HMBC spectrum of *2,2′,3-tribromo-3′,4,4′,5-tetrahydroxy-6′-methoxymethyldiphenyl-methoxymethane* (**5**) in $CD_3OD$
**Figure S11**. $^1$H-$^1$H NOESY spectrum of *2,2′,3-tribromo-3′,4,4′,5-tetrahydroxy-6′-methoxymethyldiphenyl-methoxymethane* (**5**) in $CD_3OD$
**Figure S12**. HR-MS/MS spectrum of compound **4** in negative mode
**Figure S13**. HR-MS spectrum of compound **5** in negative mode
**Figure S14**. HR-MS/MS spectrum of compound **5** in negative mode
**Figure S15**. Cytoprotective activity of compound **5** against Dox-induced cytotoxicity in H9c2 cells
**Figure S16**. Extracted Ion Chromatogram of major detected bromophenols in the crude extract of the epiphytes
**Table S1**. List of detected species and their relative contribution to epiphytic biomass

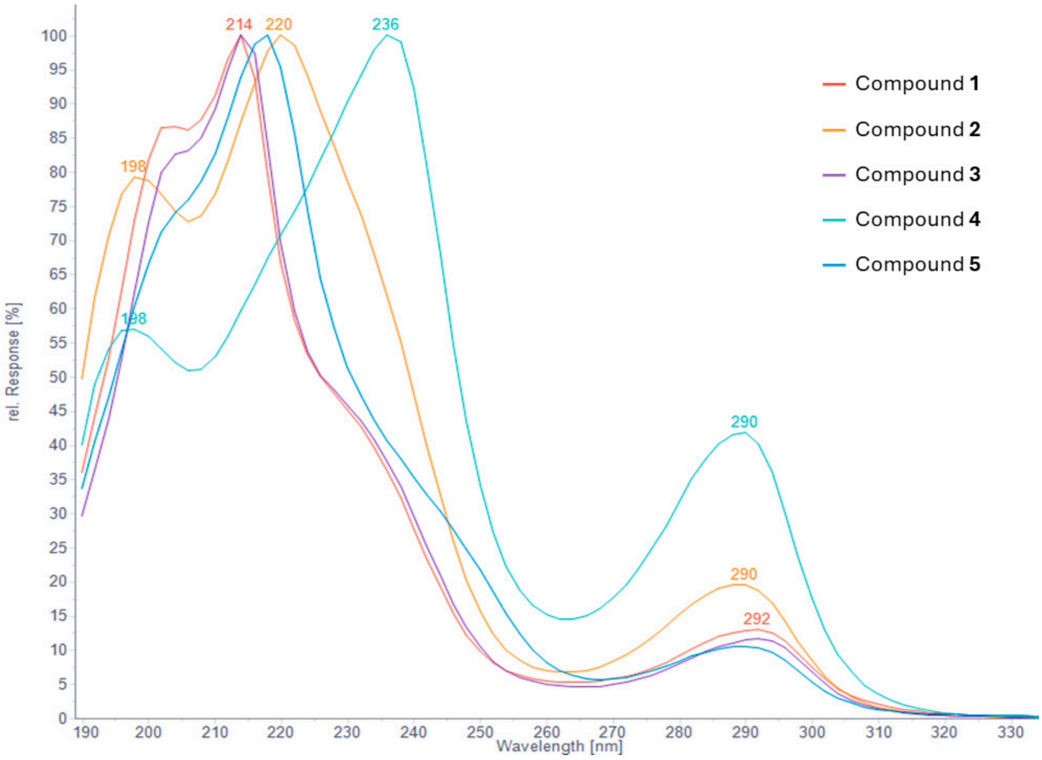


**Figure S1**. UV spectrum of compounds **1–5** in methanol

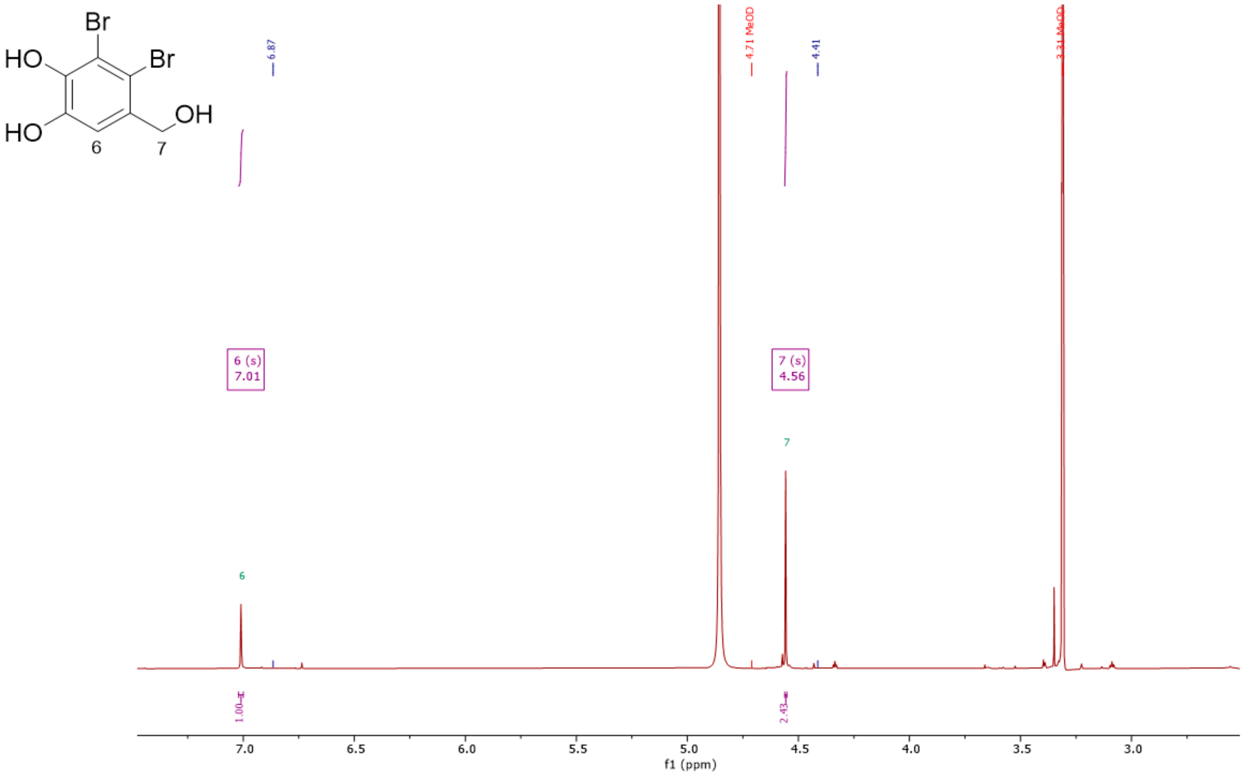


**Figure S2**. $^{1}$H NMR spectrum of *2,3-dibromo-4,5-dihydroxybenzyl alcohol* (lanosol) (**1**) in $CD_3OD$

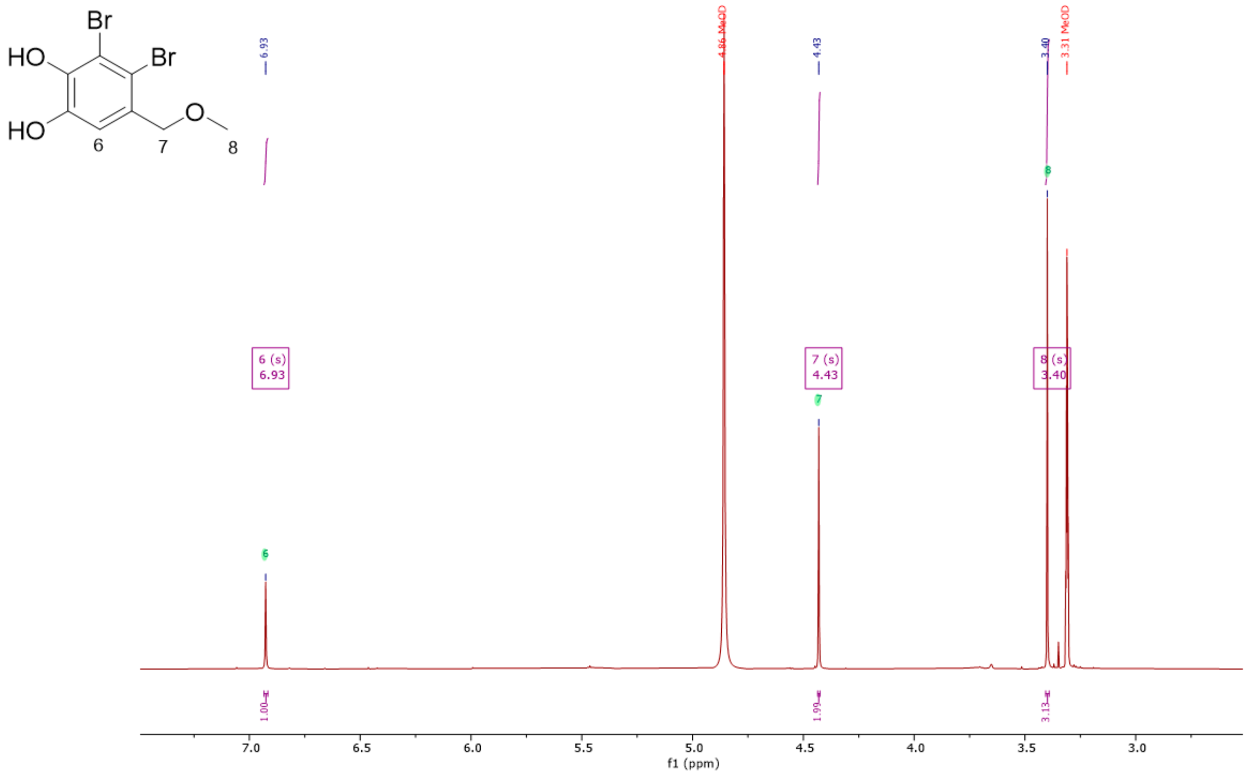


**Figure S3**. $^{1}$H NMR spectrum of *2,3-dibromo-4,5-dihydroxybenzyl methyl ether* (lanosol methyl ether) (**2**) in CD3OD

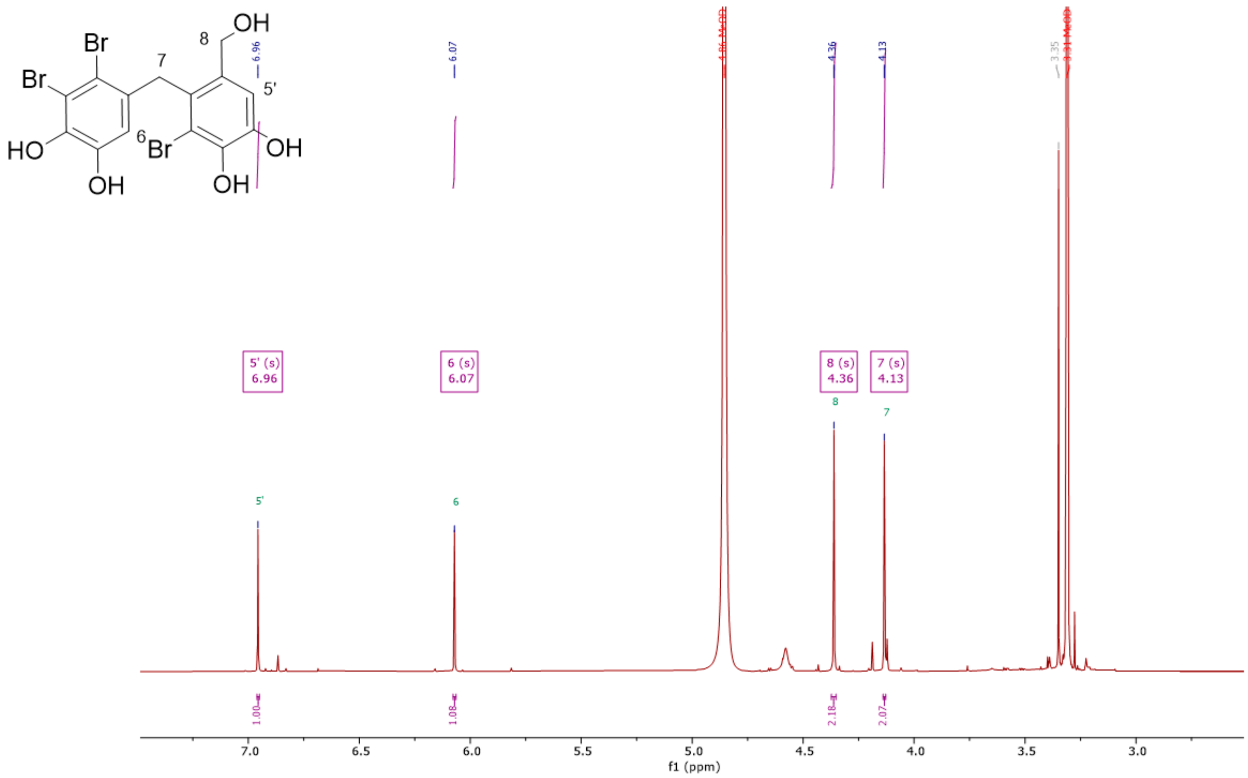


**Figure S4**. $^{1}$H NMR spectrum of *2,2′,3-tribromo-3,4,4′,5-tetrahydroxy-6′-hydroxymethyldiphenylmethane* (**3**) in CD3OD

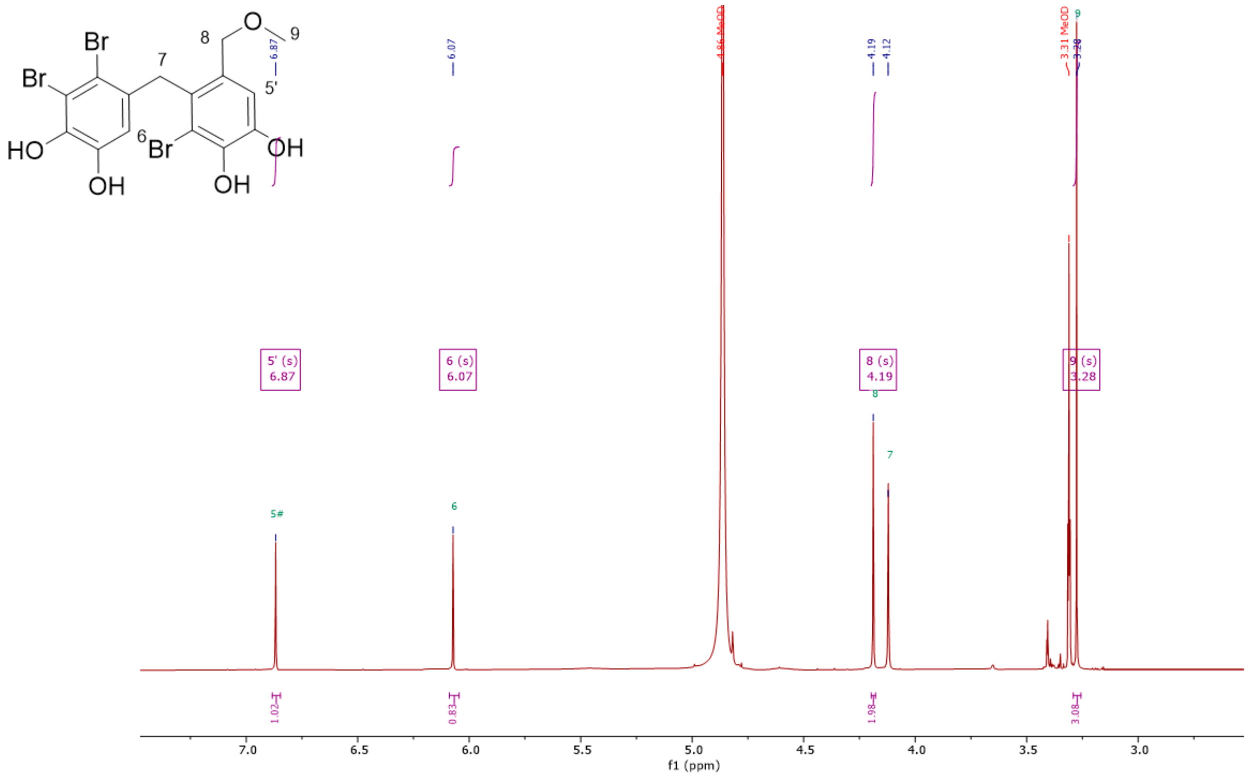


**Figure S5**. $^{1}$H NMR spectrum of *2,2′,3-tribromo-3′,4,4′,5-tetrahydroxy-6′-methoxymethyldiphenylmethane* (**4**) in $CD_3OD$

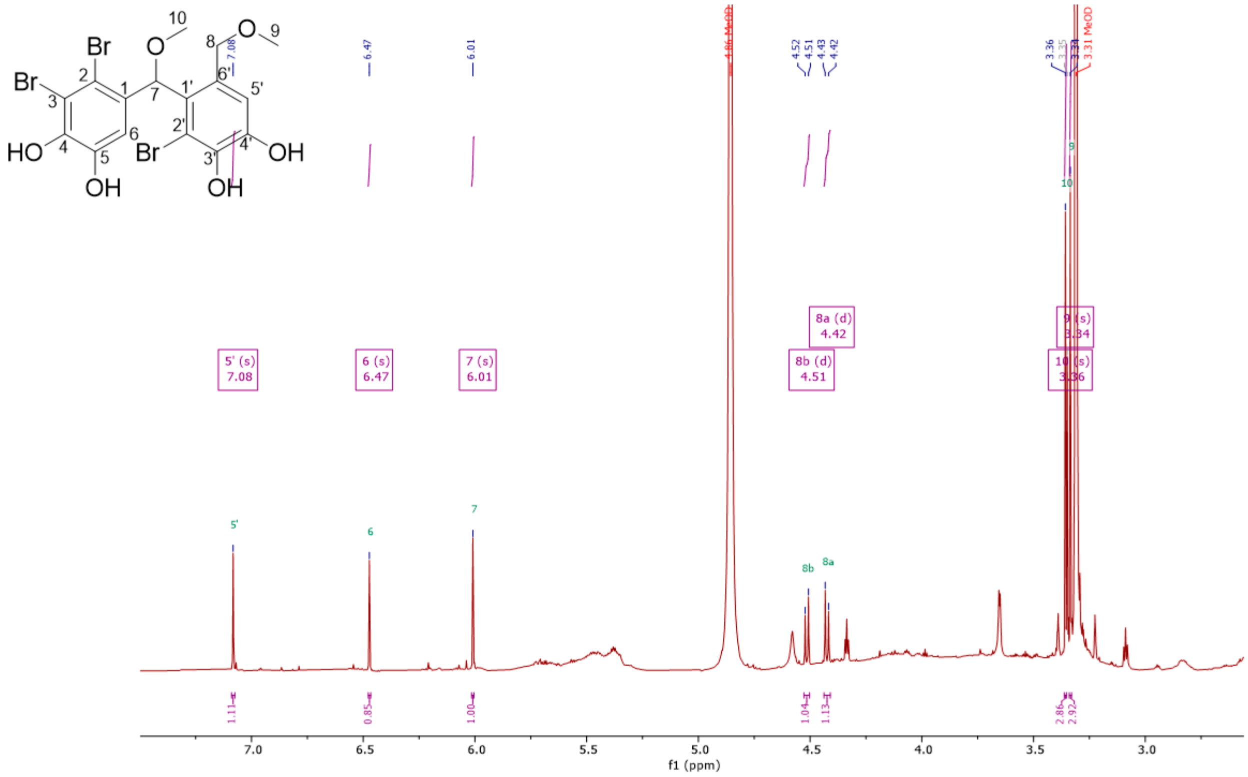


**Figure S6**. $^{1}$H NMR spectrum of *2,2′,3-tribromo-3′,4,4′,5-tetrahydroxy-6′-methoxymethyldiphenyl-methoxymethane* (**5**) in $CD_3OD$

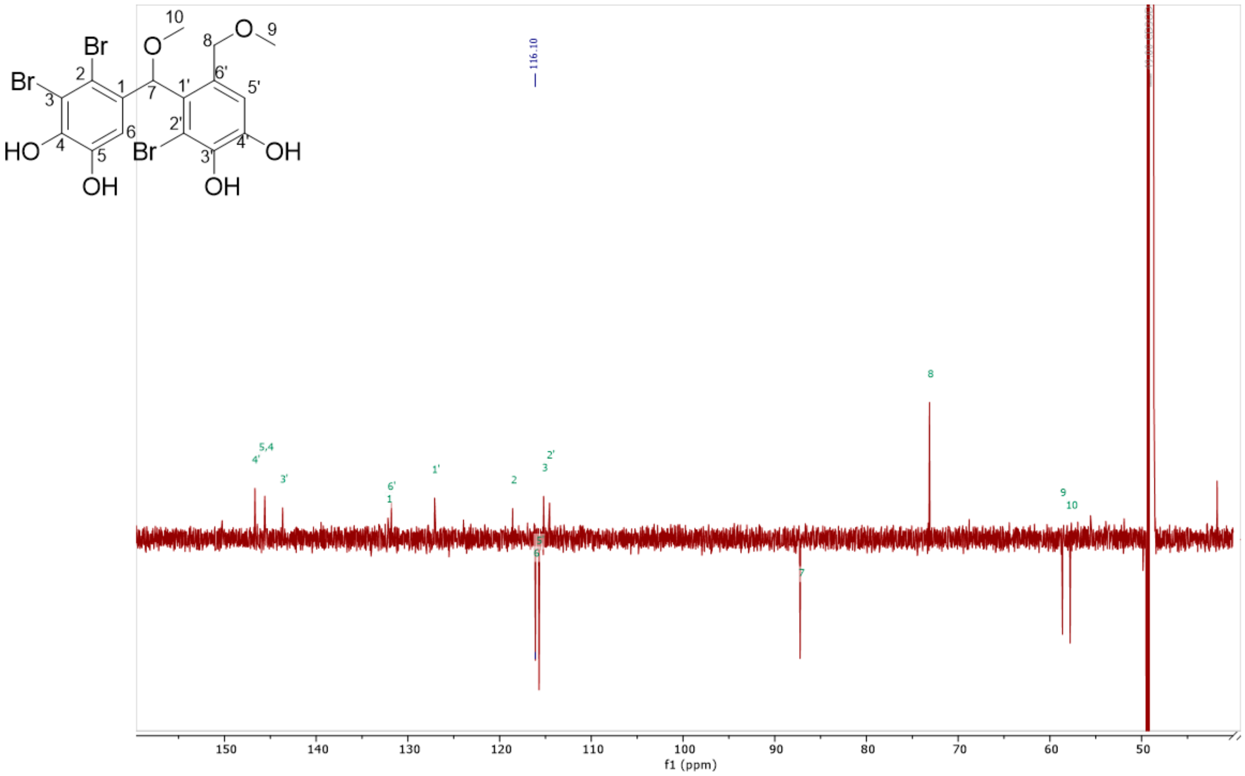


**Figure S7**. DEPT spectrum of *2,2′,3-tribromo-3′,4,4′,5-tetrahydroxy-6′-methoxymethyldiphenyl-methoxymethane* (**5**) in $CD_3OD$

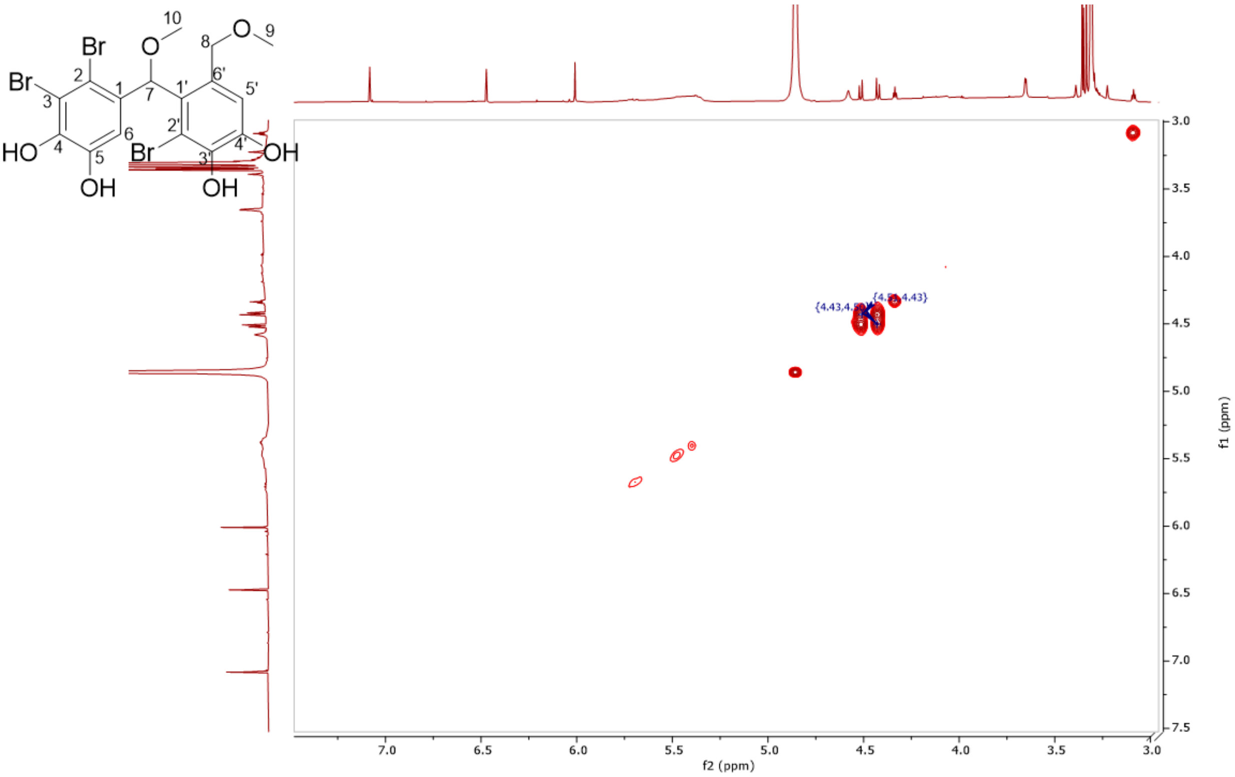


**Figure S8**. $^1H$-$^1H$ COSY spectrum of *2,2′,3-tribromo-3′,4,4′,5-tetrahydroxy-6′-methoxymethyldiphenyl-methoxymethane* (**5**) in $CD_3OD$

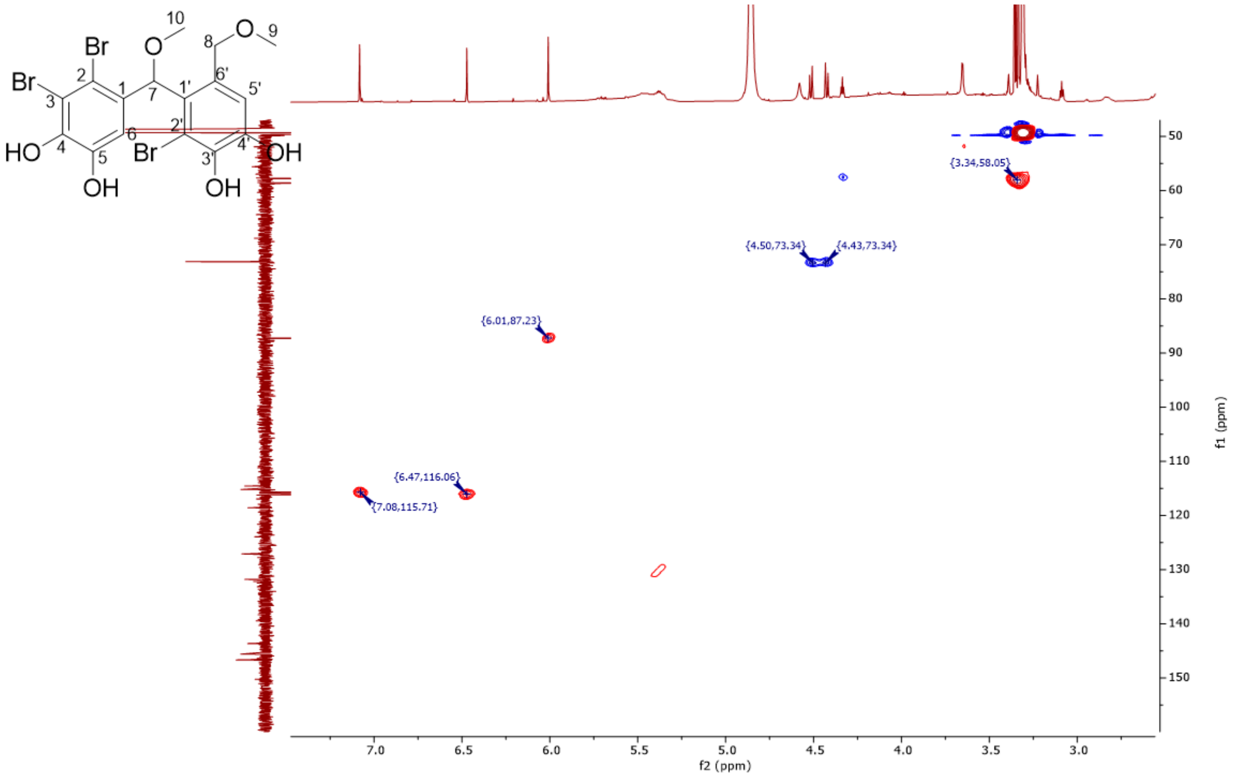


**Figure S9**. HSQC spectrum of *2,2′,3-tribromo-3′,4,4′,5-tetrahydroxy-6′-methoxymethyldiphenyl-methoxymethane* (**5**) in CD3OD

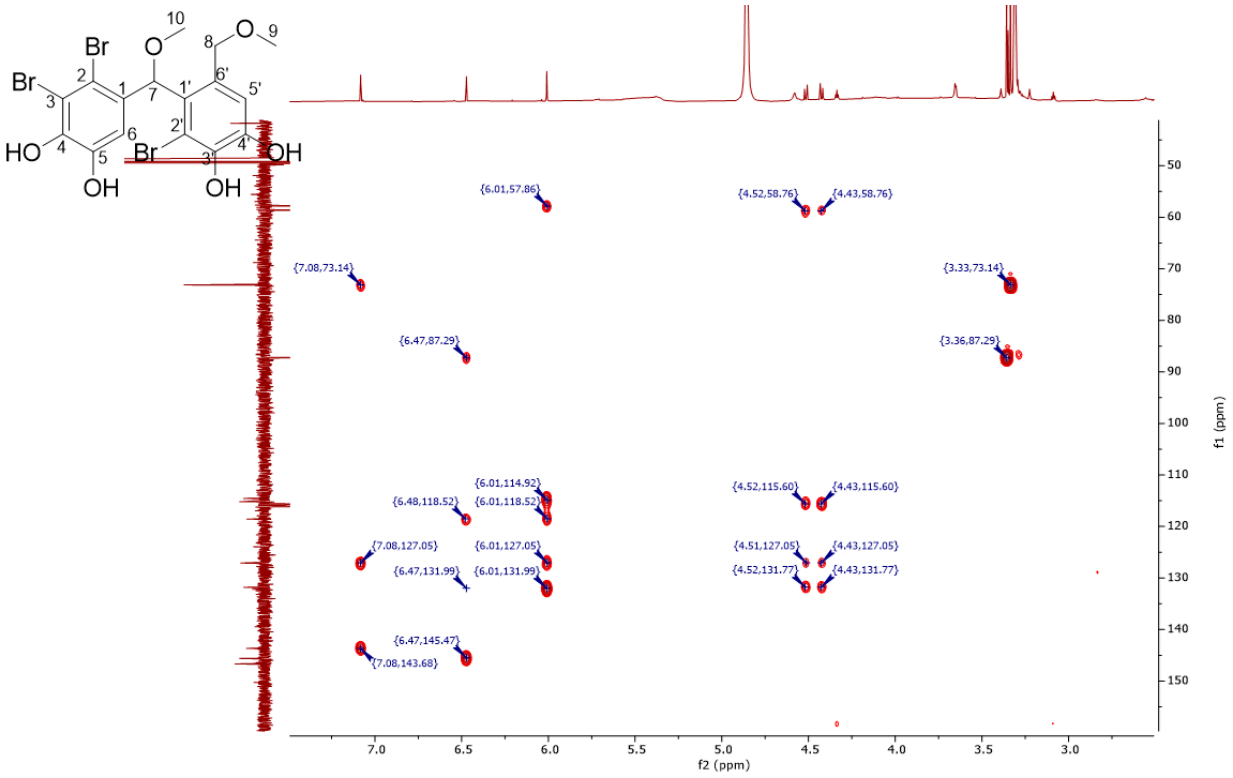


**Figure S10**. HMBC spectrum of *2,2′,3-tribromo-3′,4,4′,5-tetrahydroxy-6′-methoxymethyldiphenyl-methoxymethane* (**5**) in CD3OD

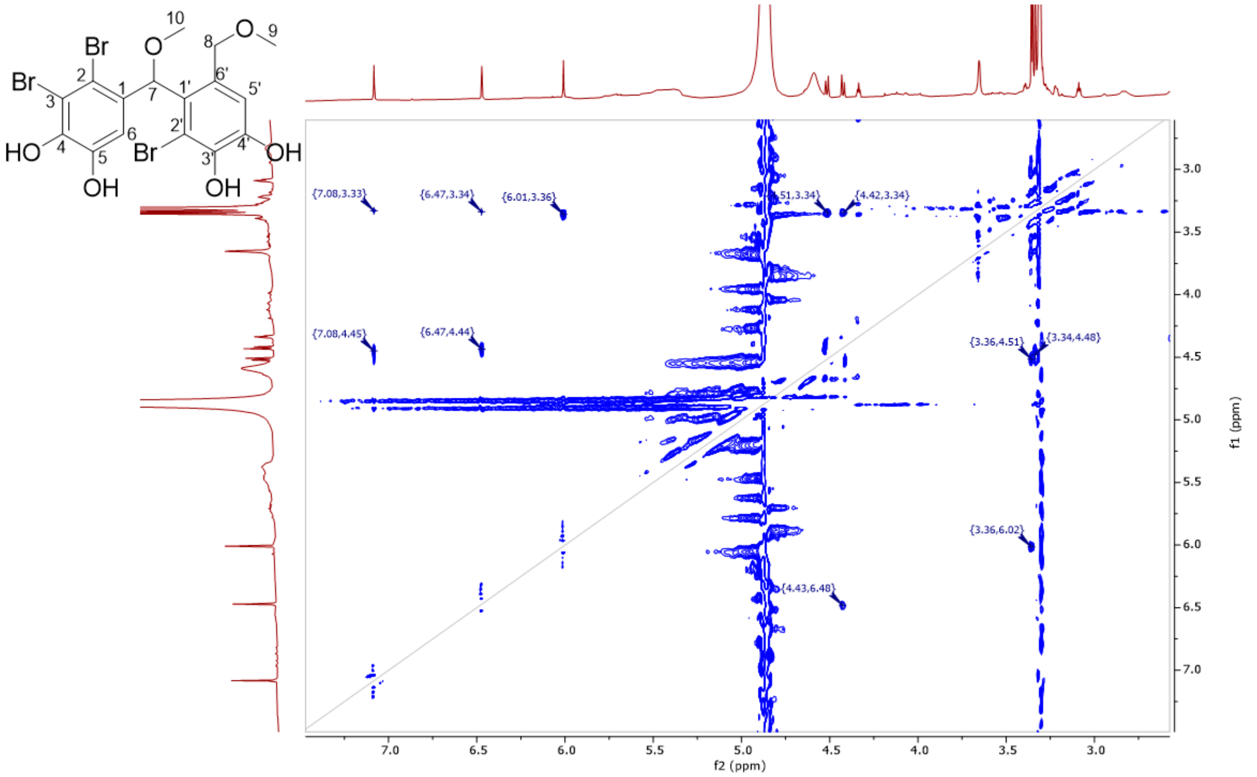


**Figure S11**. $^{1}$H-$^{1}$H NOESY spectrum of *2,2′,3-tribromo-3′,4,4′,5-tetrahydroxy-6′-methoxymethyldiphenyl-methoxymethane* (**5**) in $CD_3OD$

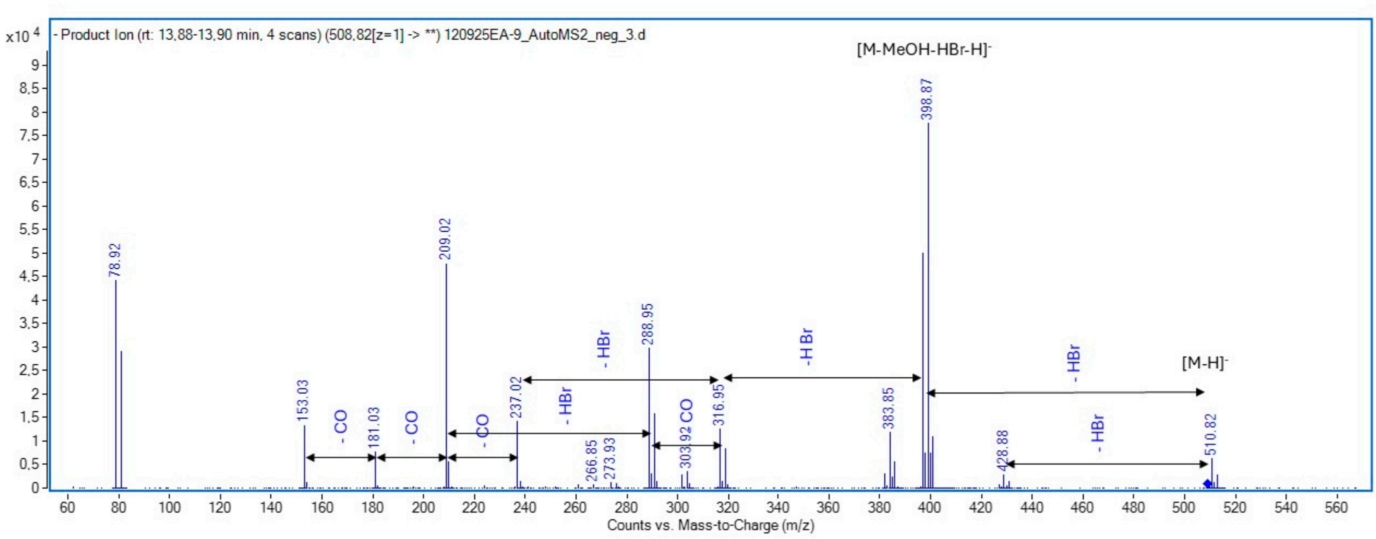


**Figure S12**. HR-MS/MS spectrum of compound **4** in negative mode

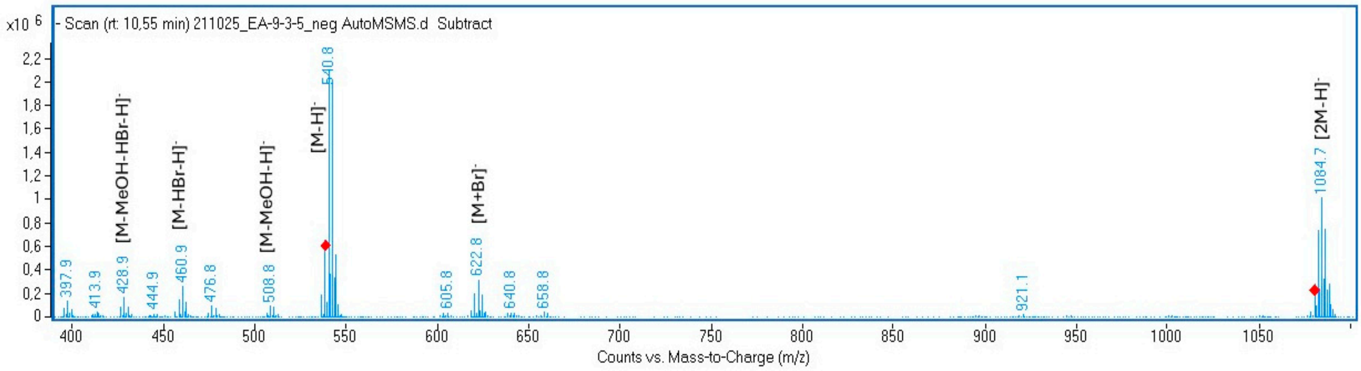


**Figure S13**. HR-MS spectrum of compound **5** in negative mode

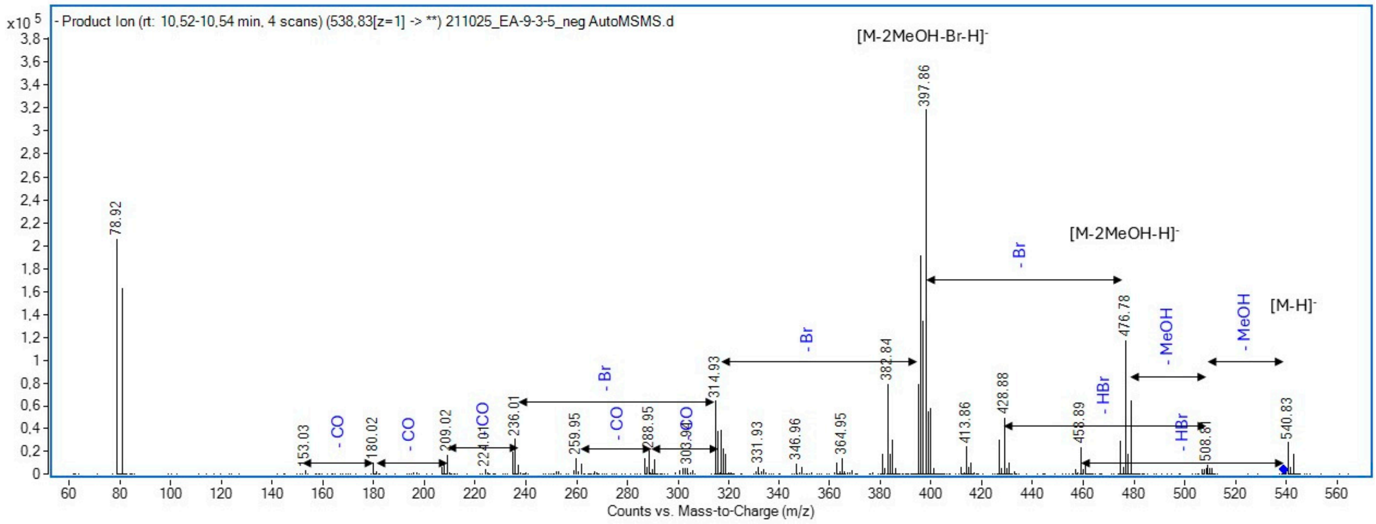


**Figure S14**. HR-MS/MS spectrum of compound **5** in negative mode

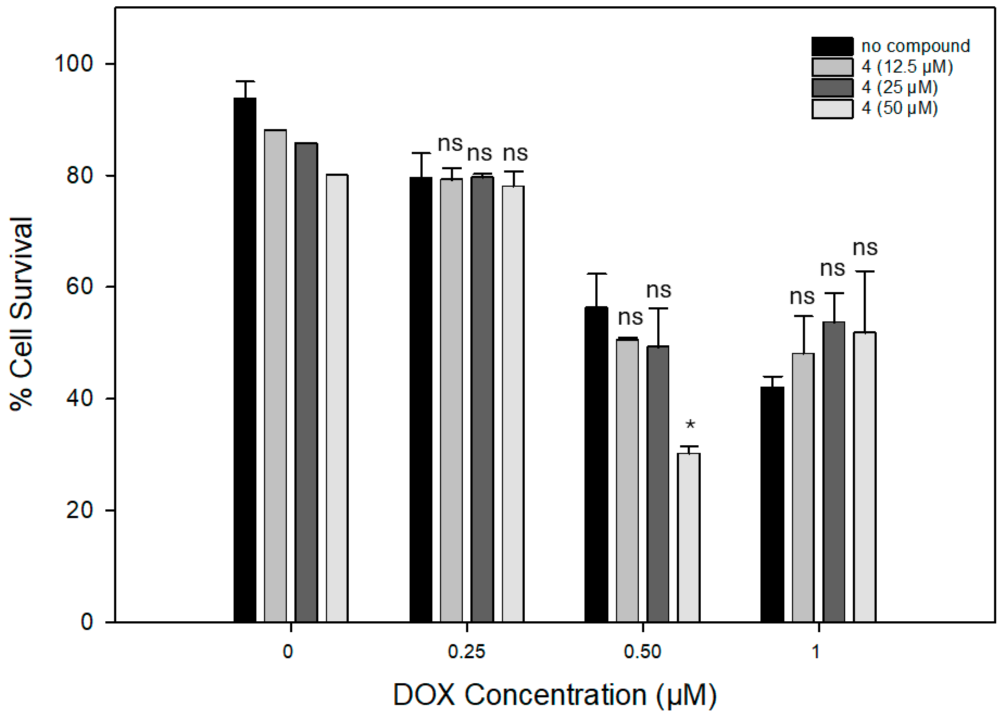


**Figure S15**. Cytoprotective activity of compound **5** against Dox-induced cytotoxicity in H9c2 cells. Cells were treated with 0, 0.25, 0.5, 1 μM doxorubicin and after 1 hour co-treated with 0 (control), 12.5, 25, 50 μM of **5** for another 24 hours. Values are expressed as mean ± standard deviation; *, $p < 0.05$, significantly different compared to the Dox-treated control; ns, not significant compared to the Dox-treated control.

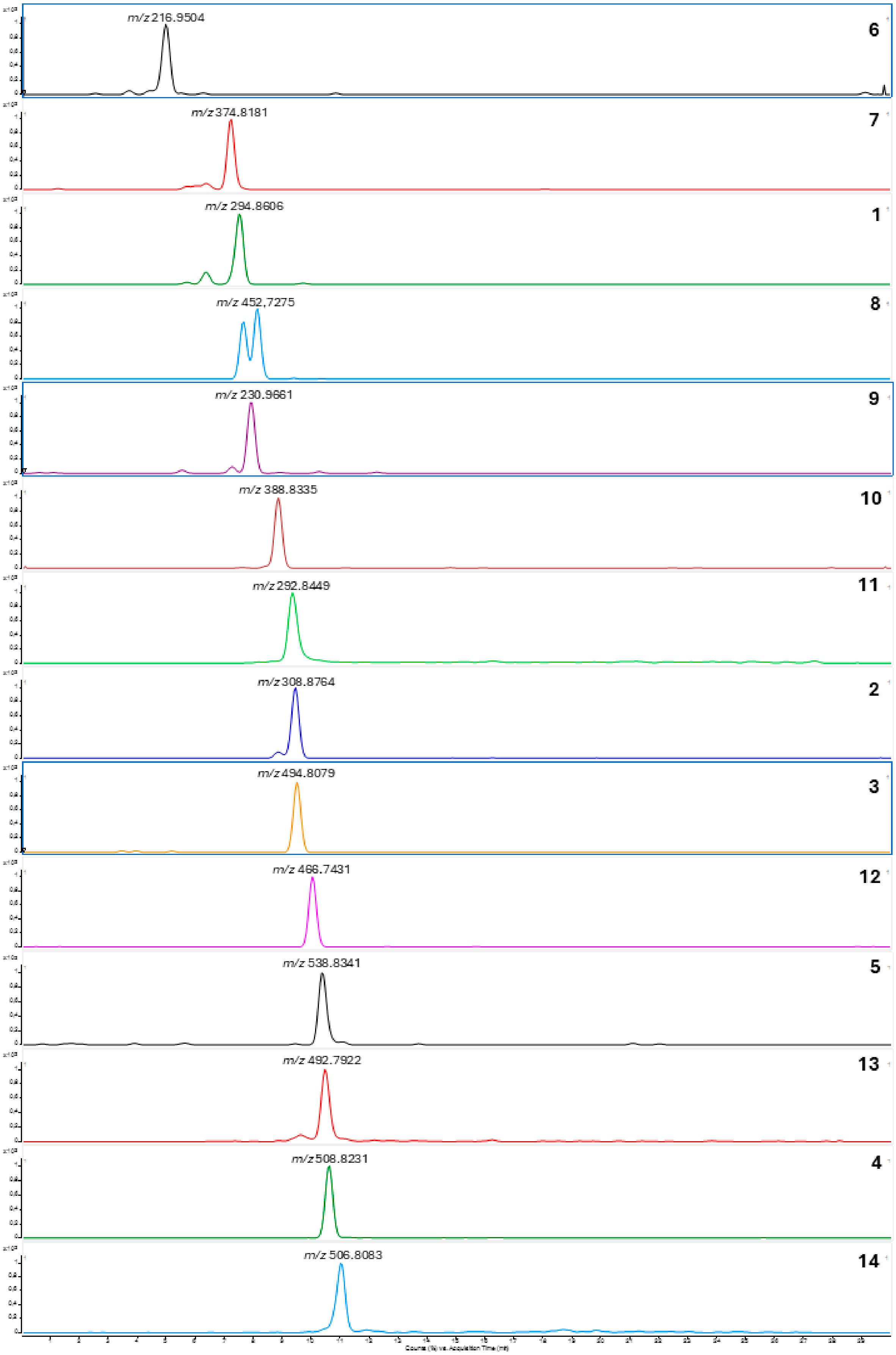


**Figure S16**. Extracted Ion Chromatogram (EIC) of major detected bromophenols **1-14** in the crude extract of the epiphytes. *m/z* value of each compound is shown on top of the peak.

**Table S1**. List of detected species and their relative contribution to epiphytic biomass. Epiphytic biomass contribution was assessed visually and reflects volumetric dominance and frequency; small filamentous taxa are therefore present but contribute minimally to total biomass Categorized as: dominant – visually forms the majority of epiphytic biomass; abundant – conspicuous and wide-spread but not dominant; common – regularly present, low volumetric contribution; occasional – present on some thalli; rare – sporadic occurrence, trace biomass

| | |
|---|---|
| **Macroalgae** | |
| Rhodophyta | |
| *Palmaria palmata* (Linnaeus) F.Weber & D.Mohr | dominant |
| *Membranoptera alata* (Hudson) Stackhouse | common |
| *Rhodomela lycopodioides* (Linnaeus) C.Agardh | abundant |
| *Ptilota gunneri* P.C.Silva, Maggs & L.M.Irvine | common |
| *Phycodrys rubens* (Linnaeus) Batters | occasional |
| *Polysiphonia spp.* | occasional |
| *Rhodomela confervoides* (Hudson) P.C.Silva | common |
| *Corallina officinalis* (Linnaeus) | rare |
| *Lomentaria articulata* (Hudson) *Lyngbye* | rare |
| *Ceramium spp.* | rare |
| Ochrophyta | |
| *Desmarestia aculeata* (Linnaeus) J.V.Lamouroux | rare |
| Juvenile *Laminaria hyperborea* (Gunnerus) Foslie | rare |
| Chlorophyta | |
| *Ulva Lactuca* (Linnaeus) | rare |
| **Low biomass epifauna** | |
| Bryozoa | |
| *Electra pilosa* (Linnaeus, 1767) | abundant |
| *Membranipora membranacea* (Linnaeus, 1767) | abundant |
| *Celleporella hyalina* (Linnaeus, 1767) | common |
| *Crisia eburnea* (Linnaeus, 1758) | occasional |
| *Crisidia cornuta* (Linnaeus, 1758) | occasional |
| Cnidaria | |
| *Dynamena pumila* (Linnaeus, 1758) | common |
| *Sertularella rugosa* (Linnaeus, 1758) | common |
| *Obelia spp.* | occasional |
| Porifera | |
| *Sycon ciliatum* (Fabricius, 1780) | rare |
